# Absence of Protein A Expression Is Associated With Higher Capsule Production in Staphylococcal Isolates

**DOI:** 10.3389/fmicb.2019.00863

**Published:** 2019-05-10

**Authors:** Tarcisio Brignoli, Andrea G. O. Manetti, Roberto Rosini, Andreas F. Haag, Vincenzo Scarlato, Fabio Bagnoli, Isabel Delany

**Affiliations:** ^1^GSK Vaccines, Siena, Italy; ^2^Department of Pharmacy and Biotechnology (FaBiT), University of Bologna, Bologna, Italy; ^3^Institute of Infection, Immunity and Inflammation, University of Glasgow, Glasgow, United Kingdom

**Keywords:** *Staphylococcus aureus*, protein A, capsule, transcription profiling, vaccines

## Abstract

*Staphylococcus aureus* is a major human pathogen, and a leading cause of soft tissue and blood stream infections. One of the causes of its success as a pathogen is the peculiar array of immune evasion factors through which the bacterium avoids host defenses, where the staphylococcal protein A (SpA) plays a major role thanks to its IgG binding activities. Moreover, SpA has recently been proposed as a promising vaccine antigen. In this study, we evaluated the expression of SpA in a collection of staphylococcal strains, about 7% of which did not express SpA (SpA^-^ strains), despite the presence of the gene. By a comparative genomic analysis, we identified that a mutation in the *spa* 5′ UTR sequence affecting the RBS is responsible for the loss of SpA in a subset of SpA^-^ strains. Using a high-throughput qRT-PCR approach on a selected panel of virulence-related genes, we identified that the SpA^-^ phenotype is associated with lower *spa* transcript levels and increased expression and production of capsule as well as other changes in the transcription of several key virulence factors. Our data suggest that the SpA^-^ phenotype has occurred in geographically distinct strains through different molecular mechanisms including both mutation, leading likely to translation alterations, and transcriptional deregulation. Furthermore, we provide evidence that SpA^-^ strains are highly susceptible to phagocytic uptake mediated by anti-capsule antibodies. These data suggest that *S. aureus* may alter its virulence factor expression pattern as an adaptation to the host or environment. Vaccination strategies targeting both SpA and capsule could therefore result in broader coverage against staphylococcal isolates than SpA alone.

## Introduction

*Staphylococcus aureus* is a Gram-positive bacterium that colonizes the human nares and skin (Kluytmans et al., 1997; Wertheim et al., 2005). It is a frequent cause of opportunistic infections that lead to a huge variety of diseases, ranging from skin and soft tissue infection to infective endocarditis and bacteremia (Tong et al., 2015). The success of this bacterium as a pathogen is directly linked to its array of virulence and immune evasion factors that enable the bacterium to escape host defenses. These include factors able to block the complement cascade, impair neutrophil chemotaxis, inhibit opsonophagocytosis and kill immune host cells (Foster, 2005). Among them, a central role is played by the Staphylococcal protein A (SpA), a cell wall-associated protein that can either be expressed on the surface of the bacterium or be secreted. SpA usually contains five repeated domains responsible for two distinct antibody binding activities. Its ability to bind IgGs through the Fc portion can prevent opsonophagocytosis by sequestering antibodies and by displaying them on the bacterial surface in an incorrect orientation (Peterson et al., 1977; Falugi et al., 2013). Moreover, SpA can also bind the V_H_3 domain of B cell receptors acting as a superantigen, thus leading to an impairment of the B cell response (Levinson et al., 1995; Silverman and Goodyear, 2006). In addition to SpA, several other staphylococcal proteins contribute to the bacterium’s ability to avoid the host immune system, and often have redundant or overlapping functions. For example, the staphylococcal immunoglobulin-binding protein (Sbi), a surface protein that also binds the Fc of immunoglobulins (Zhang et al., 1998), as well as capsular polysaccharide, Clumping Factor A (ClfA) and others, contribute to the inhibition of opsonophagocytosis, through different mechanisms (Thakker et al., 1998; Foster, 2005; Rooijakkers et al., 2005; Higgins et al., 2006; Smith et al., 2011; Kuipers et al., 2016). It is interesting to note that *S. aureus* employs multiple strategies to accomplish a single task, and considering this redundancy, it is likely that none of these factors is strictly essential for virulence. In fact, infection-related clinical isolates may naturally be deficient in a range of these factors (Peacock et al., 2002; Boyle-Vavra et al., 2015). Furthermore, animal model studies comparing virulence of isogenic single mutants showed attenuation but did not completely abolish the pathogen’s ability to infect (Patel et al., 1987; Moreillon et al., 1995; Thakker et al., 1998). One important observation is that each of these virulence factors has a particular temporal expression profile under *in vitro* growth conditions, reflecting a different expression during the distinct phases of infection (Cheung et al., 2004). As a general rule, factors involved in colonization (cell wall-associated proteins with adhesive and tissue-binding functions) are preferentially expressed during the exponential phase of an *in vitro* growth curve, while proteins involved in dissemination and spreading of the infection (exoproteins, proteases, toxins, haemolysins) are more likely to be expressed in the stationary phase (Cheung et al., 2004). This expression profile is the result of a highly complex and interconnected regulation that enables the pathogen to respond to external stimuli and environmental changes (Wang and Muir, 2016; Haag and Bagnoli, 2017). Another example of the plasticity of this pathogen resides in its ability to acquire new antibiotic resistance and virulence traits through horizontal gene transfer, making staphylococcal infections increasingly difficult to treat (Chambers and Deleo, 2009; DeLeo et al., 2010; Foster, 2017). For all these reasons, a vaccine against *S. aureus* would have an extremely beneficial impact on public health. Several attempts have been made for the development of a *S. aureus* vaccine and a number of investigative formulations have been developed in both preclinical and clinical phases of research (Fattom et al., 2004; McNeely et al., 2014). Despite the efforts, none has been licensed to date (Proctor, 2012; Spellberg and Daum, 2012; Giersing et al., 2016). In recent years, SpA has been proposed as a promising vaccine candidate, showing efficacy in passive immunization and animal models (Kim et al., 2012, 2015; Falugi et al., 2013; Varshney et al., 2018). In murine models, the vaccination with a mutated form of the protein lacking the IgG binding activities elicited high titers of SpA-specific antibodies (Kim et al., 2010). Moreover, immunization of mice with this mutated protein protected from infection in a subsequent challenge with *S. aureus* USA300, determining also an increase in the IgG titers of other staphylococcal antigens as compared with non-immunized control animals (Kim et al., 2010).

Considering the central role of SpA in staphylococcal pathogenesis and the rising interest in SpA as potential vaccine antigen, in the present study we aimed to understand the prevalence of SpA expression in a large collection of staphylococcal isolates. We identified a subset of strains carrying the gene but lacking expression to detectable levels of SpA (SpA^-^ strains). We investigated whether the absence of SpA is associated with genetic polymorphisms common to the SpA^-^ subset of strains, and whether other changes in the virulence gene expression profile are associated with the SpA^-^ phenotype, highlighting a profound interconnectedness of SpA and other immune evasion factors. Understanding how different staphylococcal immune evasion mechanisms interact and how their relative expression levels are controlled will give a deeper insight on staphylococcal pathogenesis, and support to the design of new therapies against this important human pathogen.

## Materials and Methods

### Strains and Growth Conditions

*Staphylococcus aureus* strains used in this study are listed in Supplementary Table S1. Staphylococcal strains were grown at 37°C in trypticase soy agar (TSA) plates supplemented with 5% (v/v) of sheep blood or tryptic soy broth (TSB, Difco Laboratories) at 37°C. Growth curves for each of the analyzed strains were performed to define the OD corresponding to the different growth phases. The ODs identified were OD = 0.5 for early exponential phase, OD = 2.0 for mid-exponential phase, OD = 4.0 for late exponential phase, OD = 8.0 for early stationary phase, OD = 10.0 for late stationary phase; a representative timecourse is shown in Supplementary Figure S1. *Escherichia coli* DH5α clones were grown at 37°C on LB plate or LB broth, supplemented with 100 μg ml^-1^ of ampicillin when required.

### Western Blot Analysis

*Staphylococcus aureus* cell wall-associated protein samples were prepared as already described (Corrigan et al., 2009), using liquid growth of OD_600_ = 0.6. The total protein content of the preparation was measured by the Bradford colorimetric method according to manufacturer’s instructions and equal protein amounts were loaded onto a 4–12% Bis-Tris gel, and transferred to nitrocellulose membrane using the iBlot transfer device (Thermo Fisher Scientific). For SpA detection, the membrane was incubated with SpA-specific chicken antibodies conjugated with biotin and revealed with HRP-conjugated streptavidin. Alternatively, SpA was detected as previously reported (O’Halloran et al., 2015), probing the membrane with HRP-conjugated rabbit anti-mouse IgG (1:2,000 Dako).

### Genomic DNA Extraction, Sequencing and Assembly

Genomic DNA was extracted from overnight bacterial growth using GenElute Bacterial Genomic DNA Kit (Sigma-Aldrich) according to manufacturer’s instructions. Sequencing was performed with HiSeq 2500 Sequencing System from Illumina and Paired-Ends. Paired reads were assembled using CLC genomic work bench (Qiagen). Comparative analysis was performed using BRIGG software and N315 genome as reference. Alignments of specific DNA regions were performed with MUSCLE algorithm in Geneious software (Biomatters).

### *spa* Promoter Reporter System

The promoter and the 5′UTR of *spa* gene were fused to *mCherry* in a pOS1 plasmid backbone (Supplementary Table S2). The primers (Supplementary Table S3) NWMN_0055_-266_EcoRI_F/NWMN_0055_-1_R were used to amplify a sequence of 265 bp upstream of the *spa* start codon from Newman genomic DNA, which includes the *spa* promoter, the 5′UTR region and start codon, while StamCh.R/StamCh.F primers were used to amplify the *mCherry* gene. The two amplicons were fused through fusion PCR using the complementary sequences of StamCh.F and NWMN_0055_-1_R. The resulting fusion of *spa* promoter and 5′UTR-mCherry was cloned into the pOS1 vector using *EcoRI*-*PstI* restriction sites generating the pOS1pspA. The pOS1pspA was modified to obtain the variant of the *spA* promoter lacking the RBS by whole plasmid PCR. The primers RBS_KO_pspA_F and RBS_KO_pspA_R carrying the RBS mutation were designed to anneal on the region to be modified. The PCR product was digested with DpnI and transformed into *E. coli* DH5α competent cells and several clones were sequenced to identify the plasmid containing the mutation, named pOS1pspARBS. DH5α clones carrying the empty pOS1 plasmid, the wt promoter 5’UTR-mCherry fusion and the variant containing the RBS mutation were grown overnight in LB + 100 μg/ml ampicillin, and fluorescence was excited at 584 nm and measured at 610 nm in three independent experiments.

### RNA Extraction and cDNA Synthesis

Samples for RNA extraction were collected from *S. aureus* isolates grown in liquid to the needed growth phase and stabilized using RNAprotect Bacteria Reagent (QUIAGEN, Germany). The bacterial pellet was resuspended in 1ml of Trizol reagent (Thermo Fisher Scientific) and lysed in a FastPrep^®^-24 homogenizer (MP Biomedicals). The RNA was purified using the PureLink kit (Ambion) applying an on-column DNase digestion step using the RNase-free DNase kit (QIAgen) according to the manufacturer’s instructions. Residual DNA was removed by a second DNase treatment using RQ1 DNase (Promega) followed by a second RNA purification using the PureLink kit. cDNA synthesis was performed with SuperScript First-Strand Synthesis System for RT-PCR (Thermo Fisher Scientific), using random hexamer primer for reverse transcription (RT).

### Virulence Factor Transcription Profile

The virulence factors transcript profile was assessed using the high-throughput qRT-PCR system BIOMARK HD (Fluidigm), with 84 specific TaqMan assays (Supplementary Table S4). Two Chips containing different sets of assays were used, both containing two technical replicates of the housekeeping gene *gyrB*. *gyrB* was used as housekeeping reference gene, and each assay was normalized to the mean of the *gyrB* replicates for each Chip. For the transcription kinetic profiles, all the samples were normalized to the early stationary phase using the ΔΔct method. Clusterization of genes or samples was performed using the Multiple array viewer (Mev) application with Hierarchical Clustering, Pearson correlation as distance metric.

Comparative transcriptional analysis between SpA^+^ and SpA^-^ strains was performed using GraphPad Prism 7 software as follows: the difference between mean Δct for the SpA^+^ and SpA^-^ strains was calculated for each gene at each time point; two-way ANOVA was used to establish the significance of the difference, comparing values at same time points, and correcting for multiple comparisons by Sidak correction.

### qRT-PCR

qRT-PCR was done using Platinum SYBR Green qPCR SuperMix-UDG (Invitrogen–Life Technologies) and ROX as internal control on a STRATAGEN Mx3000P QPCR system. Final data were analyzed with the ΔΔct method, normalizing samples to the expression levels of *gyrB*. The statistically significant difference between SpA^+^ and SpA^-^ strains was determined by unpaired *t*-test with Welch’s correction using GraphPad Prism 7 software.

### Capsule Immunoblot

*Staphylococcus aureus* isolates were grown to OD_600_ = 12.0, and then pelleted. The bacterial pellets were resuspended in 0.5% SDS (v/v), 5 mM DTT, 100 mM Tris (pH = 8) and treated with proteinase K for 1h at 45°C. Serial dilutions were loaded onto a nitrocellulose membrane. The membrane was blocked with PBS-Milk 10% (w/v) Tween20 0.05% (v/v) and then incubated with CP5-specific rabbit antiserum, followed by goat anti-rabbit HRP. The blots were developed using the Pierce ECL Western Blotting substrate (Thermo Fisher Scientific), the images were acquired using Chemidoc (BIO-RAD) and dot intensities were defined using Imagelab (BIO-RAD). The statistically significant difference between SpA^+^ and SpA^-^ strains dot intensities was determined by unpaired *t*-test with Welch’s correction using GraphPad Prism 7 software.

### Phagocytic Uptake Experiment

Phagocytic uptake was measured similarly to the protocol already described by Nordenfelt et al. (2012). Overnight cultures were fixed in 1% (v/v) paraformaldehyde and counted by flow cytometry. Fixed bacteria were stained for 15 min with FM-64fx stain and washed in PBS. 10^7^ stained bacteria were incubated with rabbit anti capsule serum, 1% (v/v) guinea pig complement and 10^6^ HL60 differentiated cells for 30 min in the dark. Samples were washed in PBS and fixed in 1% (v/v) paraformaldehyde for 20 min, then washed in PBS and incubated in PBS 1% (w/v) BSA and 1:100 anti-*S. aureus* polyclonal antibodies (Thermo Fisher) for 1 h. After a wash in PBS 1% (w/v) BSA, the samples were incubated in Rhodamine (TRITC) F(ab′)2 Fragment Goat Anti-Rabbit IgG (Jackson Immunoresearch), for 30 min. After a wash in PBS 1% (w/v) BSA and a second wash in PBS, the samples were resuspended in PBS and read with flow cytometry with BD LSRII (BD Biosciences). Neutrophils with only internalized bacteria were gated selecting for FM-64fx positive and TRITC negative events. For each sample, 10,000 events were analyzed using FlowJo software. In each sample, the delta geometric mean of the positive and negative populations was multiplied by the percentage of positive neutrophils, giving an estimate of the bacteria internalized for each sample. The results were analyzed using a two-way ANOVA implemented in GraphPad prism 7 software, comparing each condition -S -C within each strain to the control, and applying Sidak correction for multiple comparisons.

## Results

### Identification of a Small Subset of Strains Not Expressing SpA

**FIGURE 1:**
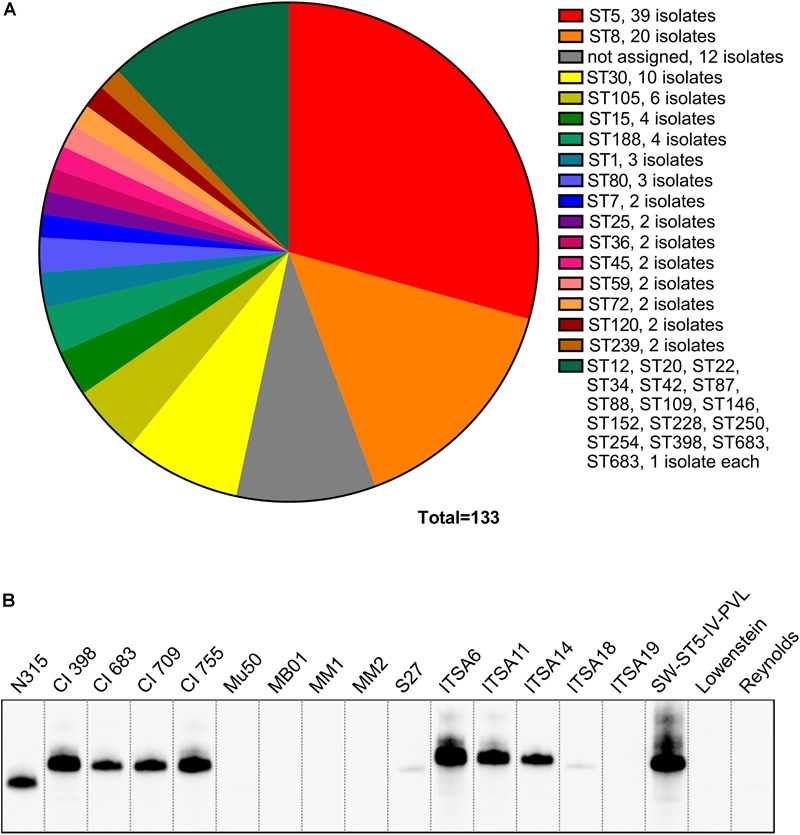
SpA expression screening in a large panel of strains. **(A)** Lineages represented in the *S. aureus* collection. The majority of the strains belong to CC5 and CC8 lineages. **(B)** SpA expression in a representative panel of strains. Western blot analysis on the SpA^-^ strains and the representative panel of SpA^+^ strains. Samples were taken from the exponential phase of *in vitro* grown cultures, SpA was detected by direct binding with rabbit anti-mouse antibody conjugated to HRP. Western blots on cell wall preparations stained with anti-SpA chicken antibody having specificity for SpA, and anti-mouse rabbit antibody conjugated to HRP capable of binding to all Ig-binding proteins, reveal similar signals (data not shown) and indicate that SpA is the only Ig-binding protein detected in these fractions.

Our first aim was to evaluate the presence of the *spa* gene and its expression in a collection of *S. aureus* strains. The collection was composed of 133 strains, including commensal and clinical isolates from diverse niches and different geographic origin as well as some lab strains, and categorized by Multilocus sequence typing (MLST) (Figure 1A). The collection included strains belonging to 33 different sequence types (ST) (Figure 1A), of which the largest portion was composed of ST5 (39 isolates) and ST8 (20 isolates), which are the most common types globally, and also ST30 (10 isolates), ST105 (6 isolates), ST15 (4 isolates), and ST 188 (4 isolates). The rest of the collection included sequence types represented by lower numbers of isolates. The collection was tested by PCR for the presence of the *spa* gene, and by Western blot for the expression of the SpA protein using a chicken anti-SpA antiserum. Although all the strains carried the *spa* locus (data not shown), nine of them resulted negative in Western blot analysis (Figure 1B). The SpA negative (SpA^-^) strains include members of clonal complex (CC) 5: Mu50 (Hiramatsu et al., 1997), four strains isolated in United States (MB01, MM1, MM2, and S27), two isolates from Italy (ITSA18 and ITSA19); and two laboratory strains belonging to CC25, Lowenstein (ATCC 49521) and Reynolds (Fournier et al., 1987). Figure 1B shows the Western blot for SpA expression in cell wall preparations in SpA^-^ strains as well as an equal number of SpA^+^ representative strains from CC5, in particular, strain N315 (Kuroda et al., 2001), four strains isolated in United States and four strains isolated in Italy. No SpA was detected in the supernatants of the SpA- strains by Western blots (data not shown), suggesting that no SpA is expressed in these strains. N315 and Mu50 are well characterized USA100 lineage strains isolated in Japan in 1982 and 1998, respectively (Hiramatsu et al., 1997). Their genomes were previously sequenced and compared, showing 96% of sequence identity (Kuroda et al., 2001; McDougal et al., 2003; Bonnstetter et al., 2007). Given their well-established characterization, Mu50 and N315 were chosen as representatives for the SpA^-^ and SpA^+^ group, respectively.

### A Deletion in the *spa* 5′UTR Affects Protein Production in a Subset of SpA^-^ Strains

**FIGURE 2:**
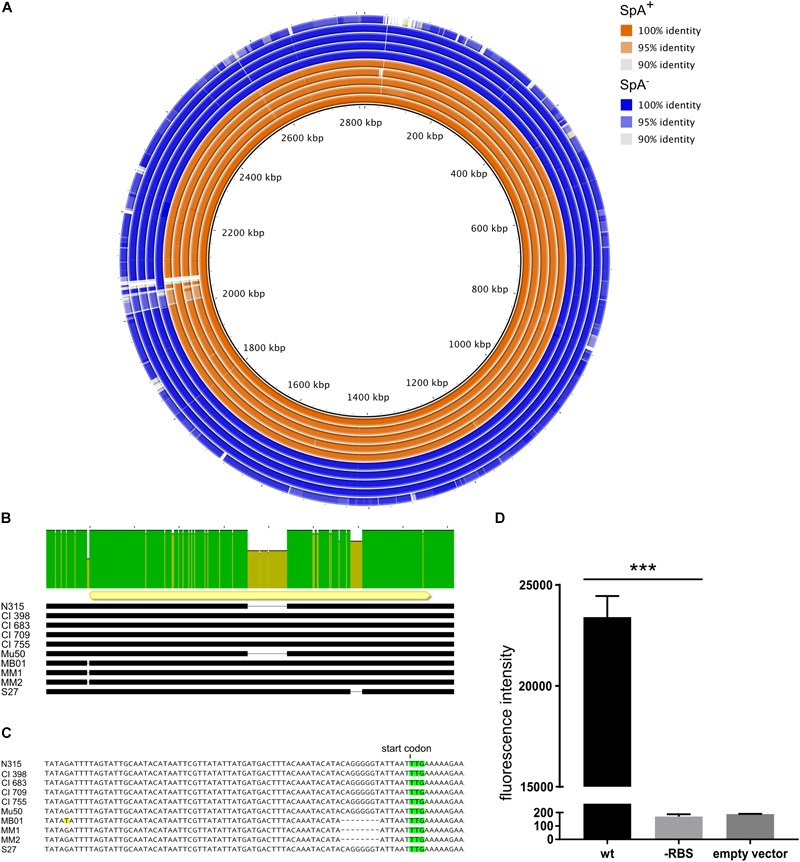
Genetic differences of SpA^+^ and SpA^-^ strains. **(A)** Comparative genomic analysis using N315 as reference (inner circle). The figure shows the genetic identity between the strains and the N315 reference strain. The SpA^+^ strains are depicted in orange, the SpA^-^ strains in blue. White gaps show regions present in the reference strain but absent in the analyzed genomes. The analyzed strains are, from the inner circle to the outer circle, N315, CI398, CI683, CI709, CI755, Mu50, MB01, MM1, MM2, and S27. **(B)** Alignment of *spa* locus in the subset of strains. The upper bar highlights the sequence identity of the *spa* locus. The yellow arrow shows the position and orientation of the *spa* coding sequence. The black bars represent the sequence in each strain and the gaps correspond to deletions. **(C)** Magnification of the region upstream of the coding sequence of *spa*. The start codon is highlighted in green and the mismatch present in the MB01 sequence is highlighted in yellow. The dashed gaps underline the deletion present in the 5′UTR region of the *spa* gene in MB01, MM1, and MM2 strains. **(D)** Effect of the 5′ UTR mutation on reporter production. The graph shows the fluorescence signal obtained from overnight growth of *E. coli* transformed with pOS1 plasmid containing either the wt *spa* promoter, or carrying the RBS mutation fused to *mCherry* reporter gene. The empty vector control shows the signal from *E. coli* strain transformed with pOS1 backbone. The graph represents three independent experiments from different clones and statistical significance was calculated by an unpaired *t*-test with Welch’s correction, ^∗∗∗^*p* < 0.001.

After the identification of the SpA^-^ phenotype in a subset of strains, we investigated the possible reasons for the loss of SpA expression. In particular, we investigated if at the level of genome sequence, we could identify a genetic basis for the SpA^-^ phenotype that could be shared by the different SpA^-^ strains. For this purpose, we analyzed the genomes of the eight United States strains (United States sub panel in Figure 1B), in addition to N315 and Mu50. Comparative genomic analysis showed that all the strains shared high sequence identity when compared to N315 (Figure 2A). As expected, Mu50 resulted with higher similarity to the N315 genome, while S27 is the isolate that carries the highest number of mutations. Most of the identified mutations are present in all the United States isolates, suggesting that these mutations are not responsible for the phenotype. It was not possible to identify a single nucleotide polymorphism or indel carried by all the SpA^-^ strains and not present in any of the SpA^+^. However, three of the SpA^-^ strains (MB01, MM1, and MM2) showed a deletion of eight nucleotides, six nucleotides upstream of the *spa* start codon (Figure 2B,C). The sequence corresponding to the deletion (CAGGGGGT) and its position make it likely to contain a Ribosome Binding Site (RBS) sequence for the *spa* gene. Further analysis on the *spa* locus showed some additional differences among the isolates (Figure 2B). The *spa* gene in Mu50 and N315 carries a deletion of 174 nucleotides, which corresponds to one IgG binding domain and whose expression results in a shorter version of the protein, as already shown in the Western blot (Figure 1B). S27 isolate carries several SNPs across the sequence as well as a shorter X variable region. Most of these mutations are silent, and none of these is responsible for an early stop codon. In order to investigate whether the deletion found in the UTR of three of the SpA^-^ strain could be responsible for their SpA^-^ phenotype, we generated two translational fusions of the *spa* promoter and 5′UTR region to an *mCherry* reporter expression system with or without the deletion. While the expression of the reporter was detected when the N315 *spa* gene upstream sequence was fused to *mCherry*, fusion of the sequence containing the deletion resulted in no fluorescence signal, confirming that this deletion is responsible for abolishing SpA expression in the three identified strains (Figure 2D). This genomic analysis showed that the genetic reasons for SpA^-^ phenotype are probably different among the different strains, but for three of them the loss of SpA expression can be attributed to a mutation in the *spa* 5′-untranslated region.

### Comparative Transcript Profiling of a Large Panel of Virulence Related Genes in SpA^+^ and SpA^-^ Strains

**FIGURE 3:**
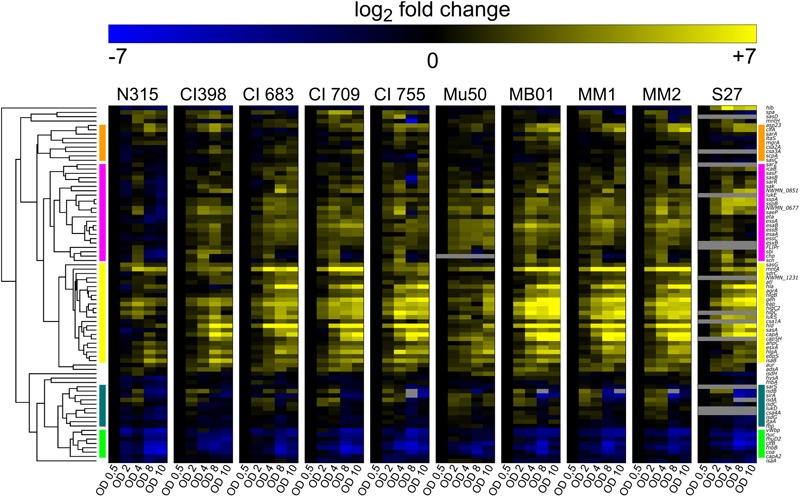
Virulence factors transcript levels variation during *in vitro* growth curve. Each column represents one assay, while each row corresponds to a sample. Values are normalized to the first growth point (early stationary phase, OD 0.5). Genes transcribed at levels lower than the first growth point are depicted in blue, up-regulated genes are in yellow. The bars on the two sides of the heatmap represent the five major clusters: cluster1 (green), cluster 2 (dark green), cluster 3 (yellow), cluster 4 (pink), cluster 5 (orange). Several assays were not able to detect target transcripts in the S27 isolate. This might have been caused either by a high degree of mismatch between the probes and the target, or the absence of the target gene.

Our genomic analysis showed that the genetic bases for the SpA^-^ phenotype are probably different among the different strains. For this reason, the SpA^-^ strains were further investigated using a high-throughput qRT PCR approach to perform comparative transcription profiling. A panel of 83 virulence related genes was selected to include factors expressed in different stages of staphylococcal infection, including adhesion, invasion and immune evasion. It is known that virulence genes exhibit *in vitro* growth phase regulation (Cheung et al., 2004), therefore, we performed the transcriptional profiling by measuring the transcript levels of the 83 virulence related genes at five representative time points of the growth curve (early, mid and late exponential phases, early and late stationary phases). The heatmap (Figure 3) shows the transcription kinetics of the selected genes in all the strains tested. All genes were clustered according to their transcription profiles through the growth curve and among the isolates. Notably, expression for the majority of the genes is increased with respect to early log phase (absorbance at 600 nm = 0.5) as indicated by the yellow predominance in the heatmap. There are five main clusters representing different transcript kinetic profiles, which are represented in Supplementary Figure S1. Cluster 1 comprises the genes whose transcription decreases during growth like *clfB* and *fnbB* (Figure 3 and Supplementary Figure S1) (Mader et al., 2016). Cluster 2 includes the genes which are upregulated in mid-exponential phase and then downregulated in stationary phase. This second cluster contains *isdA, isdB, isdC*, and *isdG* genes, which are part of the iron-regulated surface determinant pathway (Skaar and Schneewind, 2004) as well as *sirA*, which encodes for an iron-regulated lipoprotein involved in iron metabolism (Heinrichs et al., 1999), suggesting that this cluster could represent genes that respond to iron availability (Figure 3 and Supplementary Figure S1). The genes comprised in Cluster 3 are highly upregulated as growth progresses. As expected, this cluster includes genes involved in the quorum sensing system *agr*, such as *agrA* and *hld*. In particular, *agrA* encodes for the response regulator of the system, while the *hld-*coding sequence is located on the RNAIII transcript, which is the RNA effector molecule of the system (Haag and Bagnoli, 2017). Several genes known to be targets of the *agr* system are grouped in this cluster, like the capsule biosynthetic genes *capA* and *cap5H* or the alpha-haemolysin *hla* (Figure 3 and Supplementary Figure S1) (Dunman et al., 2001). The genes in cluster 4 are moderately up-regulated during growth, except for the N315 strain where they appear down-regulated (Figure 3 and Supplementary Figure S1). This is particularly evident when comparing the mean kinetic profile of N315 with the other strain (Supplementary Figure S1, cluster 4). Cluster 5 contains genes whose transcripts increase moderately during growth. The *spa* gene is not present in any of these major clusters, its transcription increases in mid exponential phase and decreases during stationary phase, while its transcription kinetics varies considerably among the isolates. The variability of *spa* transcript in SpA^-^ strains follows the upregulation of *spa* itself, which has the highest expression in the exponential phase. In Supplementary Figure S2, the *spa* transcription kinetics are shown in detail for each strain, considering the different relative steady state levels, and highlighting three different behaviors. Two of the strains with the RBS mutation (MM1 and MM2) maintained a similar profile of expression of the SpA^+^ strains, even though at lower steady state levels in comparison with SpA^+^ strains. Strains MB01 (that harbors the RBS mutation) and S27 maintained this profile only partially, as reflected by the flatter curve. Mu50 shows a completely flat trend, with similar transcript levels across the growth. This means that some of the SpA^-^ strains lost *spa* regulation during growth and expressed lower steady state *spa* transcripts.

**FIGURE 4:**
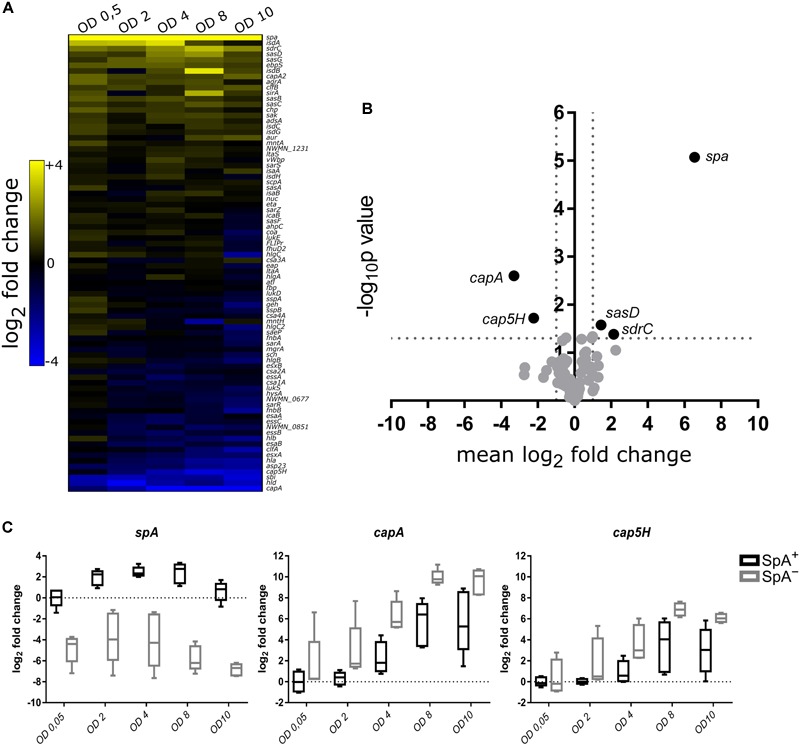
Comparative transcription profile between SpA^+^ and SpA^-^ strains. **(A)** The heatmap shows the difference between the mean RNA levels of SpA^+^ and SpA^-^ strains at each time point for each gene. The genes that are more transcribed in the SpA^+^ strains are depicted in yellow, while the ones that are more transcribed in the SpA^-^ strains are depicted in blue. **(B)** Volcano plot showing genes with significant difference in transcription. For each assay, a two-way ANOVA was performed testing the difference in transcription between SpA^+^ and SpA^-^ strains through the different growth points. Genes are displayed according to the mean difference in transcript levels between SpA^+^ and SpA^-^ strains along the entire growth, and the *p*-value measuring the associated statistical significance. On the right, with positive mean difference, the genes that are more transcribed in the SpA^+^ strains; on the left, with negative mean difference, the genes that are more transcribed in the SpA^-^ strains. **(C)** Transcription trends for *spa, capA*, and *cap5H*. The boxplots show the transcription of the three genes in the SpA^+^ and SpA^-^ strains; bars show the median values, whiskers the maximum and minimum values. The values are normalized by the mean of the SpA^+^ strains at OD 0.5. The dotted line represents the SpA^+^ mean level in early exponential phase.

To highlight the transcriptional differences between SpA^+^ and SpA^-^ strains, we calculated the difference in gene expression between the mean RNA levels of SpA^+^ and SpA^-^ strains. The genes were ranked based on their up-regulation in one or the other group and plotted in a heatmap (Figure 4A). Present at the two extremities of the heatmap are the genes that were differently expressed in each point of the growth, i.e., genes that are always upregulated in one group or the other. The center of the heatmap contains some of the genes that had different transcript levels between the SpA^+^ and SpA^-^ strains in specific growth phases. The significance of the difference in the amounts of transcripts for each gene was assessed through a two-way ANOVA, as shown by the volcano plot in Figure 4B. This analysis highlighted five genes that show significant differences in transcript levels between the two groups of strains, i.e., a twofold level or higher and a *p*-value lower than 0.05. As expected, the *spa* gene exhibits the highest upregulation in the SpA^+^ strain, while other two genes, *sdrC* and *sasD*, were upregulated in SpA^+^ strains with lower significance. Interestingly, two genes were upregulated in the SpA^-^ strain, the capsule biosynthesis related genes *capA* and *cap5H*. These two genes belong to the capsule biosynthesis operon and are the first gene of the operon (*capA*) and the first gene in the capsular polysaccharide type 5 specific region (*cap5H*), respectively (Sau et al., 1997). Notably, in the S27 isolate the *cap5H* transcript was not detected, and genome sequencing revealed a divergent sequence of the capsule biosynthetic locus, indicating that this strain most likely belongs to a different serotype. The boxplots in Figure 4C show in more detail the transcriptional changes of *spa, capA*, and *cap5H* during growth. As already reported, *spa* is upregulated during the exponential phase, while capsule biosynthetic genes are progressively upregulated during growth and mostly transcribed during stationary phase (Mader et al., 2016). With this analysis we identified five genes differentially transcribed between the SpA^+^ and SpA^-^ groups, including the upregulation of the capsule biosynthesis operon in SpA^-^ strains.

### Regulatory Network Analysis of Genes With Distinct Transcription Profiles in SpA^+^ and SpA^-^ Strains

**FIGURE 5:**
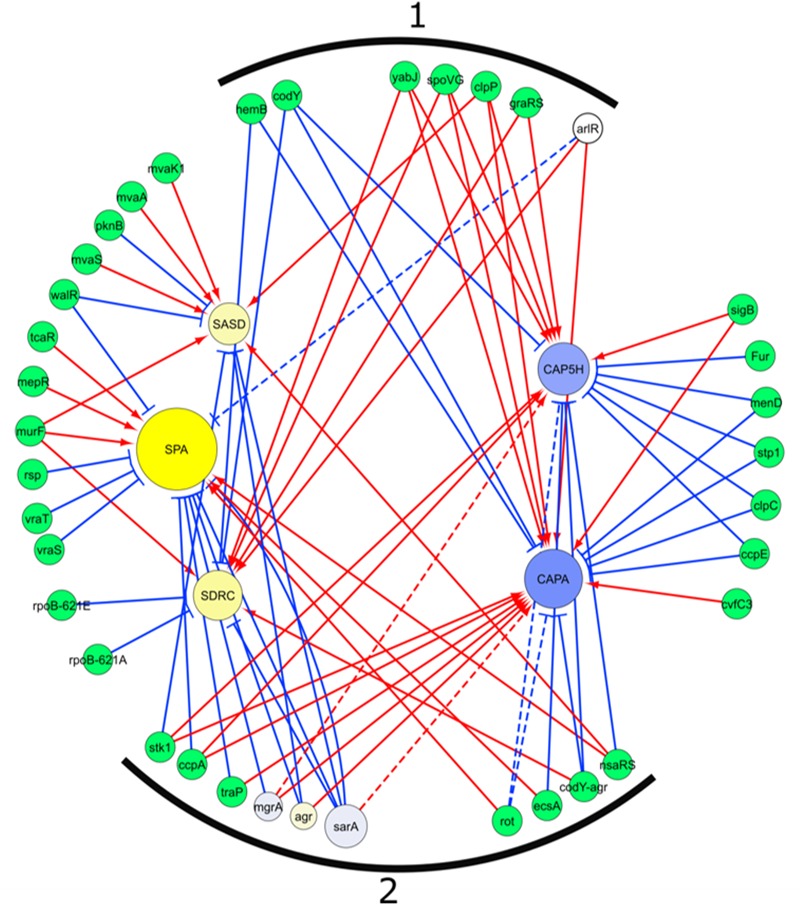
Regulatory network of genes differentially transcribed between SpA^+^ and SpA^-^. The network was extracted from the SATMD Database. The yellow circles represent genes upregulated in SpA^+^ strains, the blue circles represent the genes upregulated in the SpA^-^ strains. The color intensity represents the magnitude of upregulation in one group of strains or the other. The dimension of the circles is proportional to the -log_10_ of the *p*-value of the ANOVA testing upregulation between SpA^+^ and SpA^-^ strains. Green circles represent genes, or combination of genes, whose deletions influence the transcription of the five genes of interest. The three regulators present in the assays are depicted with the same graphic representation of the differential transcribed genes. Red arrows represent a positive regulation, blue arrows a negative regulation. Dotted lines represent interaction not found in the database, but in the literature. **(1)** Regulatory genes that influence in both groups of genes transcription, which is not consistent with transcript analysis. **(2)** Regulatory genes that exert on both groups of genes a regulation consistent with transcript data.

The known regulators of the genes with a significant difference in transcript levels between SpA^+^ and SpA^-^ strains were extracted from the SATMD *S. aureus* Transcriptome Meta-Database (Nagarajan and Elasri, 2007), and are shown in Figure 5. Three different types of genes can be identified in the regulatory network. Seven genes (Figure 5, group marked 1) are known to have similar regulatory activity on both groups of genes upregulated in SpA^+^ or SpA^-^ strains, indicating that they are unlikely involved in the SpA^-^ phenotype. Twenty genes were shown to influence the transcription of only one of the two groups of differently expressed genes, implying that one single gene of this group cannot be responsible for SpA^-^ phenotype. Ten of the regulators (*stk1, ccpA, traP, mgrA, agr, sarA, rot, ecsA, codY*-*agr*, and *nsaRS*; Figure 5, group marked 2) are reported to have an opposite regulatory activity on the genes identified as having distinct profiles in SpA^+^ and SpA^-^ group of strains. This suggests that a shift in the regulatory output of one of these regulators could result in the transcriptional differences observed in the SpA^-^ strains.

### Capsule Gene Transcription and Capsule Production Is Higher in SpA^-^ Isolates of Geographically Distinct Origin

**FIGURE 6:**
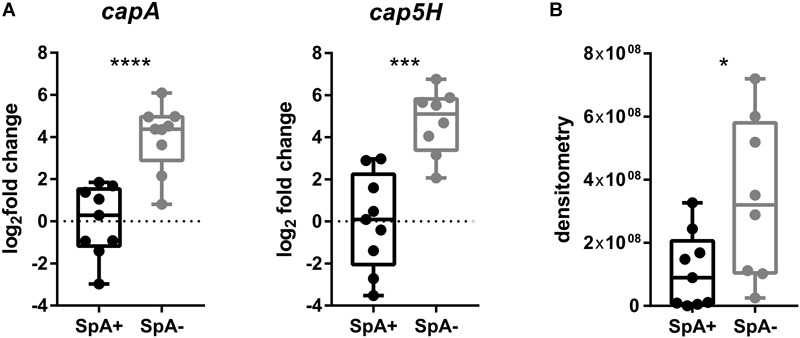
Capsule biosynthesis operon transcription and capsule production in SpA^+^ and SpA^-^ strains. **(A)** *capA* and *cap5H* transcript levels in SpA^+^ and SpA^-^ strains. **(B)** Capsule production in SpA^+^ and SpA^-^ strains. Bars indicate the medians, whiskers the maximum and minimum values. All values are relative to the mean of SpA^+^ strain levels. Statistical significance is calculated with Welch’s *t*-test, ^∗^*p* < 0.05, ^∗∗∗^*p* < 0.001, ^∗∗∗∗^*p* < 0.0001.

Having identified significant transcriptional differences in the United States subset of strains, we expanded the subset to include the remaining SpA^-^ strains as well as a control SpA^+^ group to verify the upregulation of *capA* and *cap5H* transcripts by qRT-PCR, and quantified the relative amount of capsule produced by all strains. The *capA* and *cap5H* transcript levels were quantified at a single time point by qRT-PCR. As already observed, the RNA levels of both genes were significantly higher in the SpA^-^ strains (Figure 6A). To quantify the amount of capsule produced by the SpA^-^ strains, we performed a capsule immunoblot from a single growth point (Figure 6B). The analysis showed high variability in the amount of capsule among the different strains, but the highest capsular polysaccharide amount was detected in the SpA^-^ isolates. Moreover, four of the SpA^+^ strains (N315, CI709, ITSA6, and ITSA14) produced no detectable capsule at the time point tested, while all SpA^-^ strains expressed capsule at detectable levels.

### SpA^-^ Strains Are Susceptible to Phagocytosis Mediated by Capsule-Specific Antibodies

**FIGURE 7:**
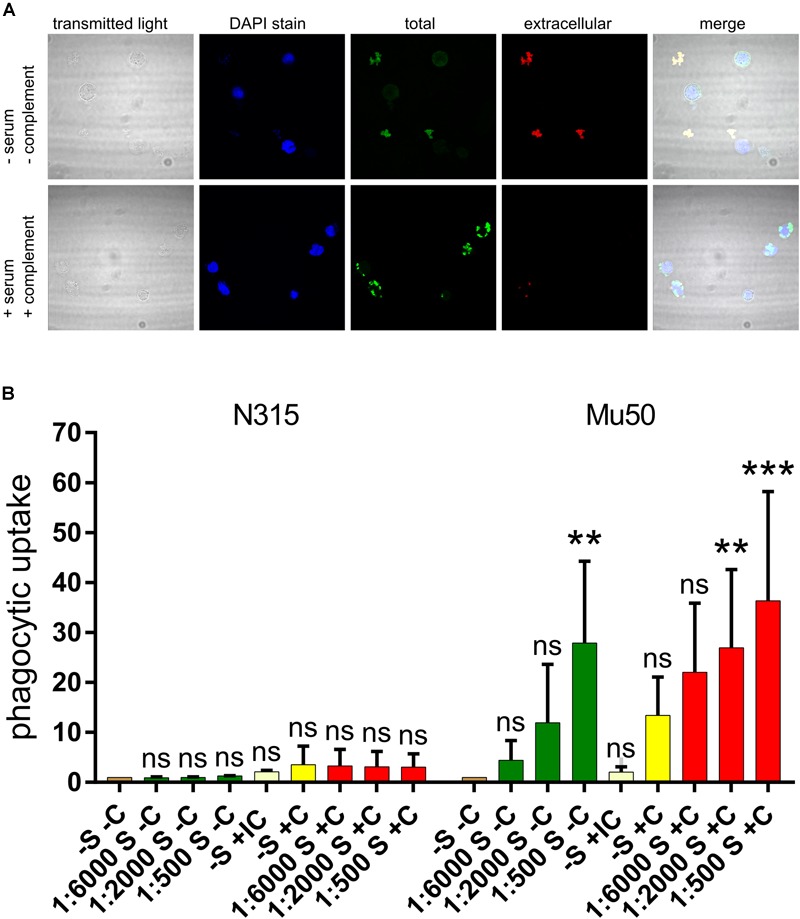
Effect of capsule specific serum on Mu50 phagocytosis. Fixed bacterial cells were incubated with differentiated HL60 cells in the presence or absence of a source of complement and capsule-specific antiserum. **(A)** Confocal microscopy visualization of phagocytosis experiments. DAPI stain binds DNA and shows neutrophil nuclei and bacterial cells (with lower intensity). Total bacteria are shown by the staining after cell permeabilization, extracellular bacteria are shown by the staining before cell permeabilization. **(B)** Effect of capsule specific antiserum on SpA^+^ and SpA^-^ phagocytosis. The graph shows the fluorescence associated to neutrophils after phagocytosis of fluorescent stained bacteria, under different conditions. The phagocytosis was performed in absence of both serum and complement (–S –C), in absence of complement and in presence of different serum dilutions (S –C), in absence of serum and in presence of inactivated complement (IC), in absence of serum and in presence of complement (–S +C), or in presence of both complement and different dilutions of serum (S +C). Each experiment was normalized by the corresponding –S –C sample. The experiment was performed in triplicate, the error bars show the standard deviations. The graph reports the results of multiple comparisons obtained with a two-way ANOVA, in which each condition was compared to the –S –C reference sample. *p*-values are indicated as ^∗∗^*p* < 0.005, ^∗∗∗^*p* < 0.0005; ns, not significant.

After we gained evidence of the fact that SpA^-^ and SpA^+^ strains produce different amounts of capsule, we explored whether the capsule produced by the SpA^-^ strains was enough to elicit neutrophil uptake in the presence of capsule-specific antibodies. N315 and Mu50 were used as SpA^-^ and SpA^+^ reference strains and a phagocytosis experiment was set up using capsule-specific rabbit antisera as described in Section “Materials and Methods.” Phagocytic uptake was visualized by confocal microscopy and quantified by Flow cytometry. Figure 7A shows representative examples of the interaction between Mu50 and neutrophils in different conditions. In the absence of complement and serum, no bacteria were associated with neutrophils. The presence of both complement and specific serum lead to the interaction of all the bacteria with the neutrophils, with almost complete phagocytic uptake. The effect of different serum dilutions and the presence or absence of complement source were tested on both Mu50 and N315 strain (Figure 7B). The minimal level of uptake observed in absence of either complement or serum was used as baseline level for both strains. In the case of Mu50, the addition of the capsule-specific serum alone induced the internalization of the bacteria in a dose-dependent manner, while with N315 the phagocytic uptake remained similar in either presence or absence of serum. The presence of complement alone determined an increased uptake for both strains, but higher in Mu50: this is probably due to a reduced susceptibility to phagocytosis in the absence of complement of the encapsulated strain (Nilsson et al., 1997). The addition of both specific serum and complement again strongly increased the uptake of the Mu50 strain but not of the N315. As N315 expresses high levels of SpA, to test that the lack of capsule-mediated opsonophagocytosis was not due to the anti-phagocytic activity of SpA, we produced an SpA mutant and determined that no difference was observed in the presence or absence of SpA in this strain (Supplementary Figure S3). With these experiments, we confirmed that SpA^-^ strains express higher quantities of capsule, and that this capsule amount is enough to elicit phagocytosis in presence of capsule specific antibodies.

## Discussion

This study aimed to investigate the expression of SpA, both virulence factor and recently proposed vaccine antigen, and its interplay with other virulence determinants. Despite the important role of SpA in staphylococcal pathogenesis, the screening of a library of strains allowed us to identify a subset of strains lacking SpA expression (SpA^-^). The molecular mechanisms that determine the SpA^-^ phenotypes were found to be different among the SpA^-^ strains and involve both RNA translation in the three SpA^-^ strains that lacked a functional *spa* RBS, and likely alterations in the transcriptional network. The different kinetic profiles of the *spa* transcript in the SpA^-^ strains, showing lower steady state levels and lower or completely absent growth phase regulation, indicate that there are several independent factors influencing *spa* transcription and causing the loss of SpA expression in these strains.

Through transcriptional profiling of a large number of virulence determinants, the major differentiating factor identified between SpA^+^ and SpA^-^ strains is the upregulation of the capsule biosynthesis operon in the SpA^-^ strains. It is important to notice that these differences are not the only transcriptional changes occurring in the SpA^-^ strains but represent a major relevant characteristic that is shared by all the SpA^-^ strains, suggesting that capsule upregulation is a common correlated adaptation. Two other genes, *sasD* and *sdrC*, encoding cell wall anchored proteins, were identified as downregulated in the SpA^-^ strains.

The regulators shared by *spa, sdrC* and *sasD* (*walR, sarA, agr*, and *nsaRS*) are known to effect the same regulatory activity on those three genes, confirming that they are part of a similar regulatory network. Conversely, the analysis of the factors influencing *spa* and capsule transcription showed that several regulators (*sarA, agr, mgrA, traP, ccpA, rot, ecsA*, and *nsaRS*) determine opposite effects on these two virulence factors, indicating that multiple systems closely control inverse regulation to balance the expression of capsule and SpA. Alterations in any one of these factors involved in maintaining this balance would lead to the inverse shift that we observed between the SpA^-^ strains.

This inverse regulation could be necessary due to the common localization of SpA and capsule on the cell surface and the somewhat redundant nature of their functions, especially in relation to evading opsonophagocytosis. Risley et al. (2007) showed how the presence of capsule on the bacterial surface masks another major surface protein Clumping factor A (ClfA) and, probably due to steric hindrance, inhibits its binding to fibrinogen. Moreover, Nanra et al. (2013) demonstrated that, in strains expressing both capsular polysaccharide and SpA, protein A does not elicit an anti-phagocytic effect toward anti-capsule specific antibodies, suggesting that capsule may interfere with SpA functions. Importantly, the fact that the SpA^-^ phenotype has occurred repeatedly through diverse mechanisms in geographically distinct locations, suggests that the loss of SpA expression may represent the response to a selective pressure under specific conditions. Moreover, multiple examples have been reported of adaptive responses where SpA and capsule expression are inversely altered. For example, lower SpA expression and higher capsule production are common phenotypic features in Vancomycin Intermediate *S. aureus* (VISA) strains (Kuroda et al., 2000; McAleese et al., 2006; Howden et al., 2008; Gardete et al., 2012; Jansen et al., 2013). VISA strains acquire resistance through several cumulative and reversible mutations, allowing the bacteria to reduce vancomycin susceptibility through an increased thickness of the cell wall (Gardete et al., 2012; Howden et al., 2014; Hu et al., 2016; McGuinness et al., 2017). Through our regulatory network analysis, the regulators identified as ‘fitting’ the observed SpA^+^/SpA^-^ regulatory effects, namely v*raSR, walRK, stk1, stp1, rpoB, clpP*, are involved in cell wall biosynthesis and often involved in VISA strains (Hu et al., 2016). While the SpA^-^ strains analyzed in this study do not exhibit significant increase in vancomycin resistance (data not shown), the overexpression of capsule in strains lacking SpA is a feature shared with that of the VISA phenotype.

Several studies based on both clinical or laboratory evidences reported that VISA strains are associated with reduced virulence (Majcherczyk et al., 2008; Horne et al., 2009; Peleg et al., 2009; Lalueza et al., 2010) and are less likely to cause acute clinical manifestation but more likely to be persistent (Cameron et al., 2011, 2017). It was proposed that the phenotypic features of VISA strains may be the consequences not only of antimicrobial treatment, but also of changes in host pathogen interactions, prompting a combined resistance and persistent phenotype (Howden et al., 2008; Gardete et al., 2012). Another staphylococcal phenotype associated to persistence is the small colony variant (SCV) phenotype (Cameron et al., 2011). In a recent comparative transcriptome study, we observed that a strong increase in capsule gene transcription and lower *spa* RNA in the SCV clones compared to parental strains is evident in their dataset (Chen et al., 2018). The presence of this common expression pattern in different clinically relevant phenotypes of *S. aureus* drives the hypothesis that the balance of SpA and capsule is a crucial feature in staphylococcal adaptation. Moreover, it is interesting to note that despite the changes in expression of these two virulence factors, their coding sequences are not affected by major mutations, suggesting that the bacterium may be able to switch from SpA^+^ to SpA^-^ phenotype and *vice versa* to adapt to different conditions. It is worth noting that the SpA^-^ isolates that we identified from 2 geographically distinct sets of clinical isolates belong to the CC5 lineage associated with HA-MRSA strains, suggesting that the adaptation to a SpA^-^ phenotype may occur preferentially in hospital acquired infections.

In the context of vaccine research, this peculiar balance is of major relevance when using prophylactic strategies targeting one of these two major virulence factors, SpA or capsule. In particular, intervention strategies targeting solely SpA may not be successful against all strains as SpA^-^ strains are found to arise independently in distinct geographical locations. Here, we show that a SpA^-^ strain is highly susceptible to the opsonic effect of anti-capsule antibodies, therefore suggesting that an intervention/vaccine strategy targeting both factors could be used for extending the coverage of SpA antigen to the broadest number of staphylococcal strains.

A vaccine strategy using capsular polysaccharide as unique antigen was already developed but failed in phase III clinical trial (Fattom et al., 2004, 2015). One of the reasons of the capsule-based vaccine failure could be the emergence of strains with no or low capsule expression, as we have shown that SpA^+^ strains may not be susceptible to capsule specific antibodies. In particular, the USA300 lineage, which in the past years became the predominant epidemic CA-MRSA strain in United States, was shown to lack the capsular polysaccharide due to conserved mutations in the capsule biosynthetic operon (Boyle-Vavra et al., 2015). The development of anti-staphylococcus therapeutic antibodies directed toward SpA is currently in clinical trials (Holland et al., 2018). The presence of a widespread number of strains that differs in virulence, and the development of several phenotypes through the adaptation to the host, suggest that a multicomponent strategy is fundamental in staphylococcal vaccine design. From this study, we have determined that SpA and capsule antigens could be targeted by either passive or active immunization as vaccine approaches to effectively target a broader range of strains with phenotypic adaptations involving different virulence determinants.

## Author Contributions

ID, FB, AH, RR, and TB designed the research. TB, AH and AM performed the research. TB, AH, RR, AM, VS, ID, and FB analyzed the data. TB and ID drafted the manuscript. All authors were involved in revising it critically for intellectual content. All authors had full access to the data and approved the manuscript before its submission.

## Conflict of Interest Statement

This study was sponsored by GlaxoSmithKline Biologicals SA which had a role in the study design, collection, analysis, interpretation of data and the writing of the manuscript as well as the decision to submit for its publication. ID, FB, RR, and AM are employees of the GSK group of companies. FB and ID report ownership of GSK stocks. FB and ID are listed as inventors on patents on vaccine candidates owned by the GSK group of companies. TB is a Ph.D. student of the University of Bologna and received a GSK Ph.D. studentship and collaborated with GSK as part of his Ph.D. training from the University of Bologna. AH was a recipient of an Intra-European Fellowship (PIEF-GA-2012-328377). The remaining author declares that the research was conducted in the absence of any commercial or financial relationships that could be construed as a potential conflict of interest.

## Supplementary Material

FIGURE S1 Kinetic profiles of the four major transcription clusters. Black lines represent the mean values of single genes and the error bars show the standard deviation among the isolates. The mean profile of the genes within the cluster is showed in pink. In cluster 4, the blue line represents the mean of the cluster in N315 strain.

FIGURE S2 *spa* transcript profiles in all the SpA^+^ and SpA^-^ strains. The black lines indicate the transcript profile of the SpA^+^ strains, while the colored ones represent the *spa* RNA levels in the SpA^-^ strains. All the values are normalized to the mean of the SpA^+^ strains in the early exponential phase. The three strains that carry the RBS mutation are highlighted in the figure legend.

FIGURE S3 Lack of capsule-mediated opsonophagocytosis in N315 background is not due to anti-phagocytic activity of SpA. The graph shows the fluorescence associated to neutrophils after phagocytosis of fluorescent stained bacteria, under different conditions. The phagocytosis was performed in absence of both serum and complement (–S –C), in absence of complement and in presence of different serum dilutions (S –C), in absence of serum and in presence of inactivated complement (IC), in absence of serum and in presence of complement (–S +C), or in presence of both complement and different dilutions of serum (S +C). Each experiment was normalized by the corresponding –S –C sample. The experiment was performed in triplicate, the error bars show the standard deviations.

TABLE S1 Strains used in this study.

TABLE S2 Plasmids used in this study.

TABLE S3 Oligonucleotides used in this study.

TABLE S4 TaqMan assays used in this study.
