# Supplementary figures and images for: Absence of Protein A Expression Is Associated With Higher Capsule Production in Staphylococcal Isolates
Source: Front Microbiol. 2019 May 10;10:863. doi: 10.3389/fmicb.2019.00863 (PMC6523524; doi:10.3389/fmicb.2019.00863)

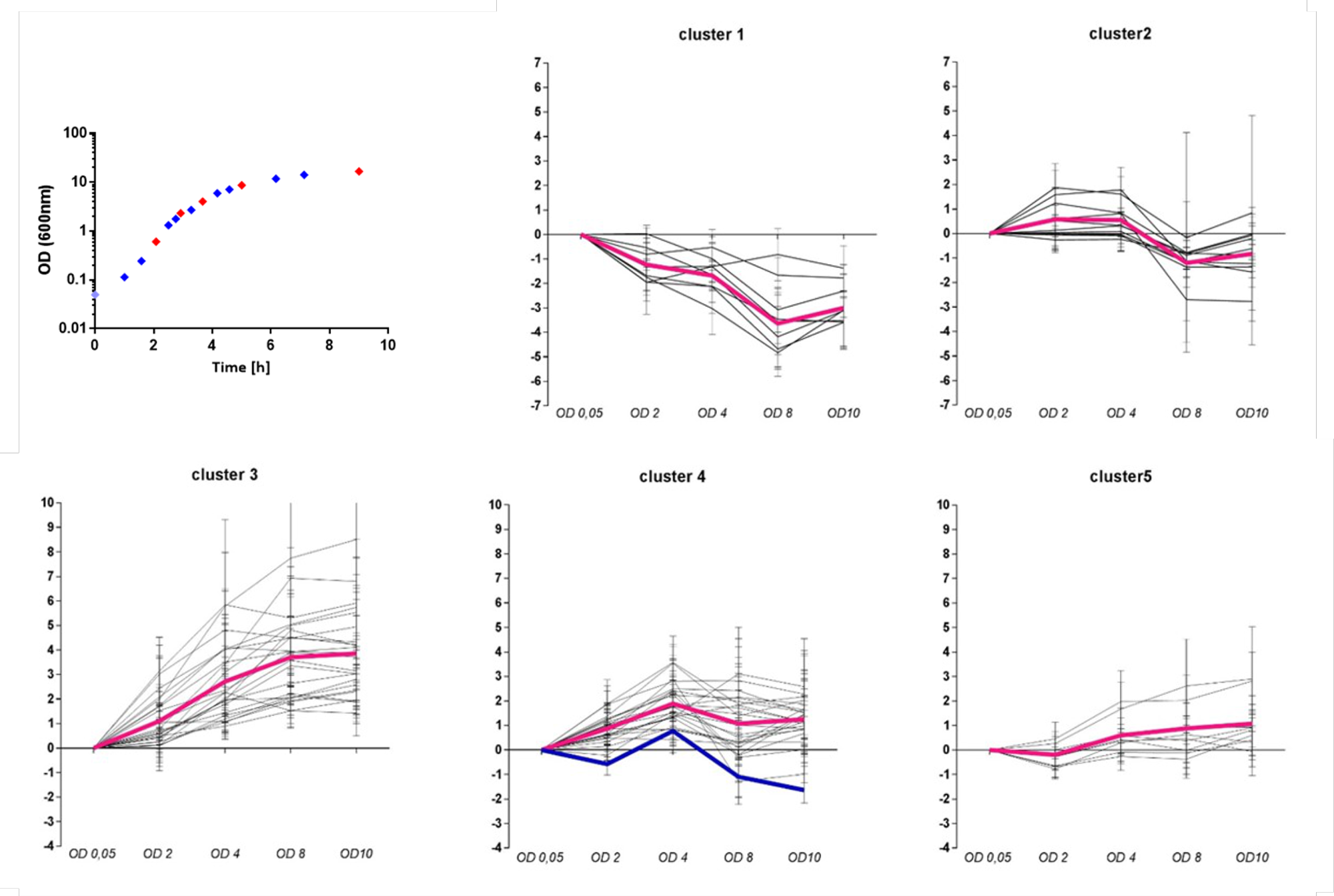

Supplement: FIGURE S1 — Kinetic profiles of the four major transcription clusters. Black lines represent the mean values of single genes and the error bars show the standard deviation among the isolates. The mean profile of the genes within the cluster is showed in pink. In cluster 4, the blue line represents the mean of the cluster in N315 strain. [file Image_1.TIF]

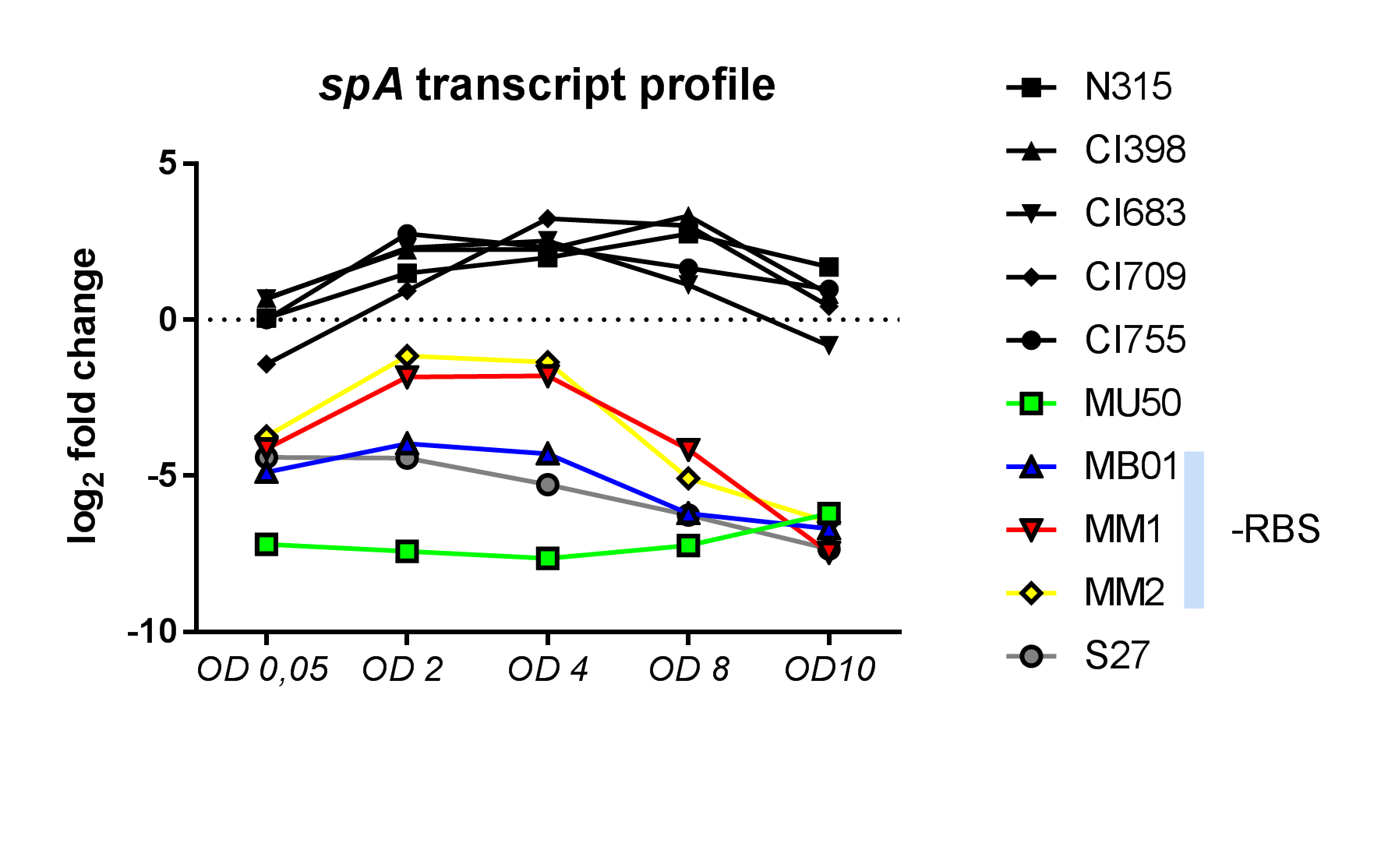

Supplement: FIGURE S2 — spa transcript profiles in all the SpA+ and SpA- strains. The black lines indicate the transcript profile of the SpA+ strains, while the colored ones represent the spa RNA levels in the SpA- strains. All the values are normalized to the mean of the SpA+ strains in the early exponential phase. The three strains that carry the RBS mutation are highlighted in the figure legend. [file Image_2.TIF]

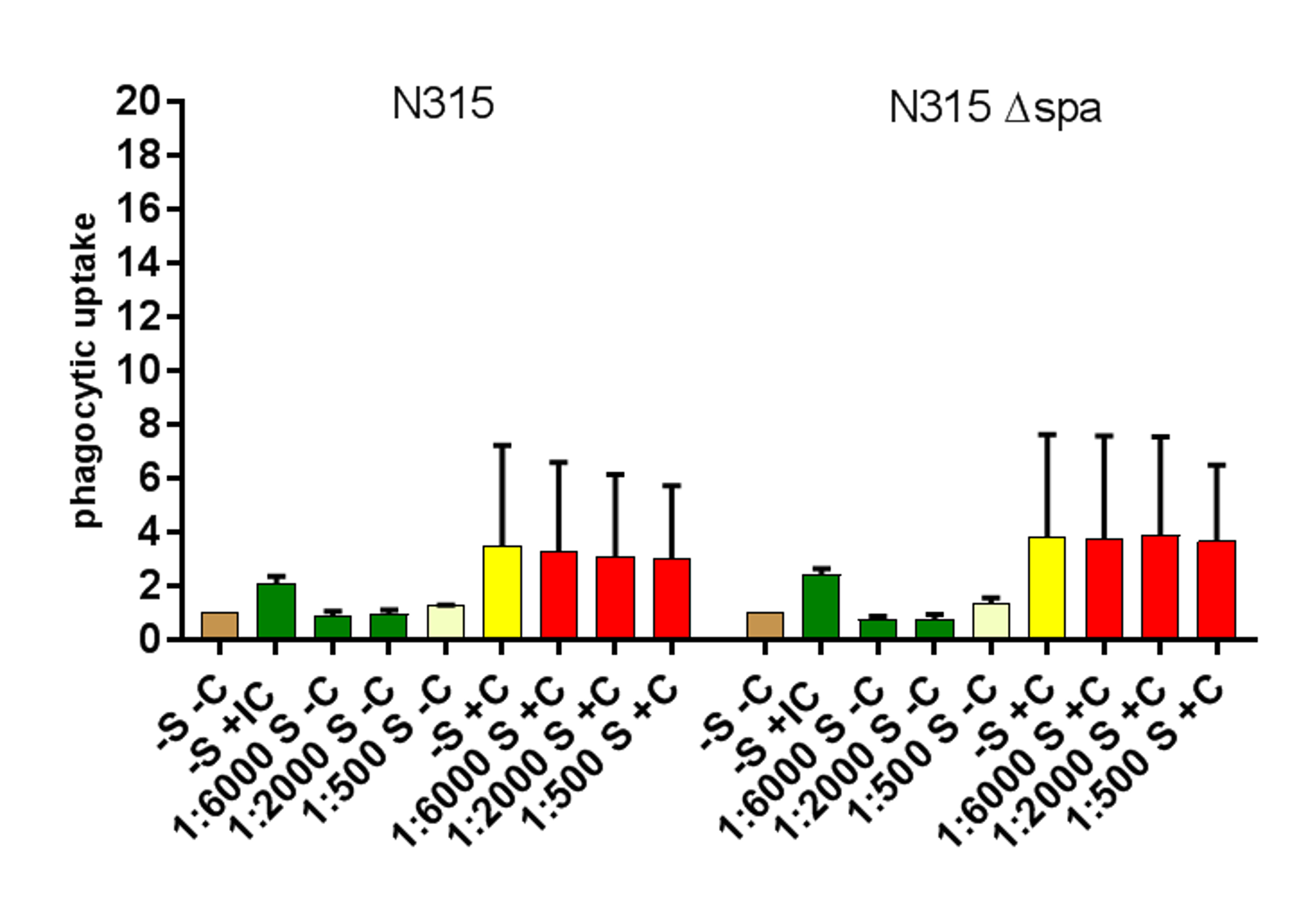

Supplement: FIGURE S3 — Lack of capsule-mediated opsonophagocytosis in N315 background is not due to anti-phagocytic activity of SpA. The graph shows the fluorescence associated to neutrophils after phagocytosis of fluorescent stained bacteria, under different conditions. The phagocytosis was performed in absence of both serum and complement (–S –C), in absence of complement and in presence of different serum dilutions (S –C), in absence of serum and in presence of inactivated complement (IC), in absence of serum and in presence of complement (–S +C), or in presence of both complement and different dilutions of serum (S +C). Each experiment was normalized by the corresponding –S –C sample. The experiment was performed in triplicate, the error bars show the standard deviations. [file Image_3.TIF]
