# Supplementary material for: Absence of Protein A Expression Is Associated With Higher Capsule Production in Staphylococcal Isolates
Source: Front Microbiol. 2019 May 10;10:863. doi: 10.3389/fmicb.2019.00863 (PMC6523524; doi:10.3389/fmicb.2019.00863)
Supplement: TABLE S1 — Strains used in this study. [file Data_Sheet_1.docx]

**Table S1. Strains used in this study.**

| **Strain** | **Description** | **Reference** | |
| --- | --- | --- | --- |
| Newman | Clinical strain, MSSA, CC8, ST254, CP5 | Baba *et al. J Bacteriol* 2008 | |
| LAC | Clinical strain, MRSA, USA300, SCCmec, IV, CC8, ST8, CP5 | Miller *et al. N Engl J Med* 2005 | |
| MW2 | Clinical strain, MRSA, USA400, SCCmec, IV, CC1, ST1, CP8 | Baba *et al. Lancet* 2002 | |
| Mu50 | Clinical strain, HA-VR-MRSA, USA100, SCCmec, II, CC5, ST5, CP5 | Hiramatsu *et al. J Antimicrob Chemother* 1997 | |
| Staph19 | Clinical strain, MRSA, SCCmec, IV, ST80, CP8 | Bagnoli *et al. PNAS* 2015 | |
| NRS216 | Clinical Strain, MSSA, ST30, CC30, CP8 | Bagnoli *et al. PNAS* 2015 | |
| Reynolds | Laboratory strain, MSSA, CC25, ST25, CP5 | Karakawa *et al. J Clin Microbiol* 1985 | |
| Wright | Laboratory strain, MSSA, CP8 | Bagnoli *et al. PNAS* 2015 | |
| Becker | Laboratory strain, MSSA, CP8 | Cook *et al. Hum Vaccin* 2009 | |
| ATCC6538 | Laboratory strain, MSSA, CC97, ST467, CP5 | Bagnoli *et al. PNAS* 2015 | |
| Lowenstein | Laboratory strain, MSSA, CC25, ST25, CP5 | Fattom *et al.* *Infect Immun* 1990 | |
| BD1686 | Clinical strain, HA-MRSA, USA100, CC5, ST5, CP5 | Bagnoli *et al. PNAS* 2015 | |
| BD1534 | Clinical strain, HA-MRSA, USA200, CC30, ST36, CP8 | Bagnoli *et al. PNAS* 2015 | |
| BD1449 | Clinical strain, CA-MRSA, USA1000, CC59, ST59, CP8 | Bagnoli *et al. PNAS* 2015 | |
| NRS382 | Clinical strain, HA-MRSA, USA100, CC5, ST5, CP5 | Bagnoli *et al. PNAS* 2015 | |
| NRS248 | Clinical strain, MRSA, CC1, ST1, CP8 | Bagnoli *et al. PNAS* 2015 | |
| NRS252 | Clinical strain, MSSA, CC30, ST30, CP8 | Bagnoli *et al. PNAS* 2015 | |
| Staph 15 | Clinical strain, CA-MRSA, ST30, CC30, CP8 | Bagnoli *et al. PNAS* 2015 | |
| Staph 17 | Clinical strain, CA-MRSA, ST8, CC8, CP5 | Bagnoli *et al. PNAS* 2015 | |
| Staph 18 | Clinical strain, CA-MRSA, ST8, CC8, CP5 | Bagnoli *et al. PNAS* 2015 | |
| Staph 21 | Clinical strain, CA-MRSA, SCCmec, IV, ST80, CP8 | Bagnoli *et al. PNAS* 2015 | |
| MSSA 94 ISS | Clinical strain, MSSA, CC22, ST22, CP5 | Bagnoli *et al. PNAS* 2015 | |
| IT-SA1 | Clinical strain from infective endocarditis, CC30, ST30, CP8 | Rindi *et al. Eur J Clin Invest* 2006 | |
| IT-SA2 | Clinical strain from infective endocarditis, CP8 | Rindi *et al. Eur J Clin Invest* 2006 | |
| IT-SA3 | Clinical strain from infective endocarditis, CC101, ST101, CP8 | Rindi *et al. Eur J Clin Invest* 2006 | |
| IT-SA4 | Clinical strain from infective endocarditis, CC30, ST34, CP8 | Rindi *et al. Eur J Clin Invest* 2006 | |
| IT-SA5 | Clinical strain from infective endocarditis, CC8, ST8, CP5 | Rindi *et al. Eur J Clin Invest* 2006 | |
| IT-SA6 | Clinical strain from infective endocarditis, CC5, ST5, CP5 | Rindi *et al. Eur J Clin Invest* 2006 | |
| IT-SA7 | Clinical strain from infective endocarditis, CC20, ST20, CP5 | Rindi *et al. Eur J Clin Invest* 2006 | |
| IT-SA8 | Clinical strain from infective endocarditis, CC15, ST15, CP8 | Rindi *et al. Eur J Clin Invest* 2006 | |
| IT-SA9 | Clinical strain from infective endocarditis, CC121, ST120, CP8 | Rindi *et al. Eur J Clin Invest* 2006 | |
| IT-SA10 | Clinical strain from infective endocarditis, CC15, ST15, CP8 | Rindi *et al. Eur J Clin Invest* 2006 | |
| IT-SA11 | Clinical strain from infective endocarditis, CC5, ST5, CP8 | Rindi *et al. Eur J Clin Invest* 2006 | |
| IT-SA12 | Clinical strain from infective endocarditis, CC72, ST72, CP5 | Rindi *et al. Eur J Clin Invest* 2006 | |
| IT-SA14 | Clinical strain from infective endocarditis, CC5, ST5, CP5 | Rindi *et al. Eur J Clin Invest* 2006 | |
| IT-SA15 | Clinical strain from infective endocarditis, CC45, ST45, CP8 | Rindi *et al. Eur J Clin Invest* 2006 | |
| IT-SA16 | Clinical strain from infective endocarditis, CC72, ST72, CP8 | Rindi *et al. Eur J Clin Invest* 2006 | |
| IT-SA17 | Clinical strain from infective endocarditis, CC121, ST120, CP8 | Rindi *et al. Eur J Clin Invest* 2006 | |
| IT-SA18 | Clinical strain from infective endocarditis, CC5, ST5, CP5 | Rindi *et al. Eur J Clin Invest* 2006 |  |
| IT-SA19 | Clinical strain from infective endocarditis, CC5, ST5, CP5 | Rindi *et al. Eur J Clin Invest* 2006 |  |
| SW-ST239-III | Clinical strain, MRSA, SCCmec, III, CC8, ST239, CP8 | Bagnoli *et al. PNAS* 2015 |  |
| SW-ST80-IV-PVL | Clinical strain, MRSA, SCCmec, IV, pvl+, ST80, CP8 | Bagnoli *et al. PNAS* 2015 |  |
| SW-ST5-IV-PVL | Clinical strain, MRSA, SCCmec, IV, pvl+, CC5, ST5, CP5 | Bagnoli *et al. PNAS* 2015 |  |
| SW-ST30-PVL | Clinical strain, MRSA, pvl+, CC30, ST30 CP8 | Bagnoli *et al. PNAS* 2015 |  |
| SW-ST398 | Clinical strain, MRSA, CC398, ST398, CP5 | Bagnoli *et al. PNAS* 2015 |  |
| SW-ST228-I | Clinical strain, MRSA, CC5, ST288, CP5 | Bagnoli *et al. PNAS* 2015 |  |
| SW-ST8-IV | Clinical strain, MRSA, CC8, ST8, CP5 | Bagnoli *et al. PNAS* 2015 |  |
| SW-ST88 | Clinical strain, MRSA, CC88, ST88, CP8 | Bagnoli *et al. PNAS* 2015 |  |
| SW-ST45 | Clinical strain, MRSA, CC45, ST45, CP8 | Bagnoli *et al. PNAS* 2015 |  |
| SW-ST42 | Clinical strain, MSSA, Singleton, ST42, CP8 | Bagnoli *et al. PNAS* 2015 |  |
| SW-ST152 | Clinical strain, MRSA, CC152, ST152, CP5 | Bagnoli *et al. PNAS* 2015 |  |
| SW-ST59 | Clinical strain, MRSA, CC59, ST59, CP8 | Bagnoli *et al. PNAS* 2015 |  |
| BW01 | Clinical strain, HA, USA 100, ST5 | this study |  |
| CF Serology 1 | Clinical strain, HA, USA 100, ST30 | this study |  |
| CF Serology 10 | Clinical strain, HA, USA 100, ST8 | this study |  |
| CF Serology 11 | Clinical strain, HA, USA 100, ST188 | this study |  |
| CF Serology 16 | Clinical strain, HA, USA 100, ST15 | this study |  |
| CF Serology 2 | Clinical strain, HA, USA 100, ST5 | this study |  |
| CF Serology 22 | Clinical strain, , USA 100 | this study |  |
| CF Serology 25 | Clinical strain, HA, USA 100, ST109 | this study |  |
| CF Serology 26 | Clinical strain, HA, USA 100, ST8 | this study |  |
| CF Serology 29 | Clinical strain, , USA 100, ST5 | this study |  |
| CF Serology 30 | Clinical strain, HA, USA 100, ST5 | this study |  |
| CF Serology 31 | Clinical strain, , USA 100, ST10 | this study |  |
| CF Serology 32 | Clinical strain, HA, USA 100, ST5 | this study |  |
| CF Serology 33 | Clinical strain, , USA 100, ST5 | this study |  |
| CF Serology 34 | Clinical strain, HA, USA 100, ST5 | this study |  |
| CF Serology 5 | Clinical strain, , USA 100, ST30 | this study |  |
| CI 1434 | Clinical strain, HA, USA 100, ST5 | this study |  |
| CI 1438 | Clinical strain, HA, USA 100, ST5 | this study |  |
| CI 1492 | Clinical strain, HA, USA 100, ST5 | this study |  |
| CI 334 | Clinical strain, HA, USA 100, ST5 | this study |  |
| CI 394 | Clinical strain, HA, USA 100, ST5 | this study |  |
| CI 398 | Clinical strain, HA, USA 100, ST5 | this study |  |
| CI 482 | Clinical strain, HA, USA 100, ST5 | this study |  |
| CI 683 | Clinical strain, HA, USA 100, ST105 | this study |  |
| CI 697 | Clinical strain, CA, USA 100, ST5 | this study |  |
| CI 709 | Clinical strain, HA, USA 100, ST5 | this study |  |
| CI 755 | Clinical strain, HA, USA 100, ST5 | this study |  |
| CI 846 | Clinical strain, HA, USA 100, ST5 | this study |  |
| CO01 | Clinical strain, HA, USA 100, ST5 | this study |  |
| CR01 | Clinical strain, HA, USA 100, ST8 | this study |  |
| DB1 | Clinical strain, CA, USA 300, ST8 | this study |  |
| DH1 | Clinical strain, CA, USA 300, ST683 | this study |  |
| DL1 | Clinical strain, HA, USA 300, ST105 | this study |  |
| DL2 | Clinical strain, HA, USA 300, ST105 | this study |  |
| DLBAL-L | Clinical strain, HA, USA 300, ST105 | this study |  |
| DLBAL-R | Clinical strain, HA, USA 300, ST105 | this study |  |
| GM1 | Clinical strain, HA, USA 300, ST5 | this study |  |
| GM2 | Clinical strain, HA, USA 300, ST5 | this study |  |
| JMH1 | Clinical strain, HA, USA 300, ST146 | this study |  |
| KC1 | Clinical strain, HA, USA 300, ST8 | this study |  |
| LH01_Hem | Clinical strain, HA, USA 300 | this study |  |
| M 299 LIM | Clinical strain, CA, USA 700, ST5 | this study |  |
| M016 LIM | Clinical strain, CA, USA 700, ST | this study |  |
| MB01 | Clinical strain, CA, USA 700, ST5 | this study |  |
| MB02_Hem | Clinical strain, CA, USA 700 | this study |  |
| MG | Clinical strain, CA, USA 700, ST8 | this study |  |
| MM01 | Clinical strain, CA, USA 700, ST8 | this study |  |
| MM1 | Clinical strain, CA, USA 700, ST5 | this study |  |
| MM2 | Clinical strain, CA, USA 700, ST5 | this study |  |
| RF01 | Clinical strain, CA, USA 1100, ST8 | this study |  |
| RT1 | Clinical strain, CA, USA 1100, ST105 | this study |  |
| Serology 12 | Clinical strain, CA, USA 1100, ST12 | this study |  |
| Serology 16 | Clinical strain, CA, USA 300, ST5 | this study |  |
| Serology 2 | Clinical strain, CA, USA 100, ST8 | this study |  |
| Serology 21 | Clinical strain, CA, USA 100, ST8 | this study |  |
| Serology 22 | Clinical strain, CA, USA 500, ST30 | this study |  |
| Serology 23 | Clinical strain, CA, USA 200, ST8 | this study |  |
| Serology 27 | Clinical strain, CA, USA 500, ST87 | this study |  |
| Serology 28 | Clinical strain, CA, USA 100, ST8 | this study |  |
| Serology 36 | Clinical strain, CA, USA 300 | this study |  |
| SG4NQ | Clinical strain, CA, USA 200, ST5 | this study |  |
| SG6NH | Clinical strain, HA, USA 200, ST7 | this study |  |
| SG6NM | Clinical strain, HA, USA 400/700, ST7 | this study |  |
| SG9NS | Clinical strain, HA, USA 200, ST15 | this study |  |
| SGBAL | Clinical strain, HA, USA 300, ST5 | this study |  |
| TH1 | Clinical strain, HA, USA 400, ST8 | this study |  |
| TR10NK | Clinical strain, HA, USA 900, ST | this study |  |
| TR10NL | Clinical strain, HA, USA 1000, ST | this study |  |
| WB4NT | Clinical strain, HA, USA 400, ST30 | this study |  |
| WB9nm | Clinical strain, HA, USA 300/200, ST188 | this study |  |
| WB9NO | Clinical strain, HA, USA 800, ST188 | this study |  |
| WB9Nq | Clinical strain, HA, USA 400, ST188 | this study |  |
| RN4220 | Laboratory strain, restriction-deficient mutant | Kreiswirth *et al. Nature* 1983 |  |

**Table S2. Plasmids used in this study.**

| **Name** | **Description** | **Antobitic resistance** | **Reference** |
| --- | --- | --- | --- |
| pOS1 | Ori+ for Gram-positive strains; Ori− (pBR322) plasmid replication in Gram-negative bacteria | chloramphenicol/ ampicillin | Schneewind *et al.*EMBO J 1993 |
| pOS1pspA | pOS1 derivative harboring *spa* promoter and 5'UTR fusion with mCherry reporter gene | chloramphenicol/ ampicillin | this study |
| pOS1pspARBS | pOS1pspA derivative carriyng the 5'UTR variant lacking the RBS | chloramphenicol/ ampicillin | this study |
|  |  |  |  |
|  |  |  |  |

**Table S3. Oligonucleotides used in this study.**

| **Name** | **Sequence** | **Restriction**  **site** | | **Application** | **Reference** |
| --- | --- | --- | --- | --- | --- |
| StamCh.F | ATGGTGTCAAAAGGTGAAGAAGATAATATG | N/A | amplification for fusion to Staph promoters | | this study |
| StamCh.R | GCTTGGctgcagTTATTTGTATAATTC | PstI | amplification for fusion to Staph promoters | | this study |
| NWMN_0055_-266_EcoRI_F | ATCCGGgaattcGAAATTAAACCTCAGCACATTCAAAG | EcoRI | *spa* promoter for mCherry fusion | | this study |
| NWMN_0055_-1_R | CTTCTTCACCTTTTGACACCATATTAATACCCCCTGTATGTATTTGTAAAG | N/A | *spa* promoter for mCherry fusion | | this study |
| RBS_KO_pspA_F | ACAAATACATAATTAATATGGTGTCAAAAGGTGAAGAAG | N/A | RBS deletion pOS1pspA | | this study |
| RBS_KO_pspA_R | CATATTAATTATGTATTTGTAAAGTCATCATAATATAACG | N/A | RBS deletion pOS1pspA | | this study |
| capA_rt_F | TATCAACATCCAAGTTAAAAGTGG | N/A | qRT PCR *capA* | | this study |
| capA_rt_R | TCCAATATAACTGTATTCACCAATG | N/A | qRT PCR *capA* | | this study |
| cap5H_rt | GAAAAACCAGTCCTCTAAAGAATC | N/A | qRT PCR *cap5H* | | this study |
| cap5H_rt | GGTGCGACTTTAACTGCTG | N/A | qRT PCR *cap5H* | | this study |

**Table S4. TaqMan assays used in this study.**

| **Assay name** | **Newman gene** | **Oligo function** | **Sequence** | **Function-notes** |
| --- | --- | --- | --- | --- |
| *adsA* | NWMN_0022 | Forward primer | CATCACTTTCACCACGAATGTTTG | adenosine synthase A |
|  |  | Reverse primer | GCGTCTAACATTAAATCAGGCTTTTC |  |
|  |  | Probe | TTACATACAAATGATATCC |  |
| *agrA* | NWMN_1946 | Forward primer | CTCGCAACTGATAATCCTTATGAGG | AgrA - accessory gene regulator A |
|  |  | Reverse primer | GTAACGAAAATAATGTTACCAACTGGG |  |
|  |  | Probe | GATATTCAACTTTCAACTG |  |
| *ahpC* | NWMN_0372 | Forward primer | GAAATCTTACCATTTACAGCGCAAG | Alkyl hydroperoxide reductase subunit C |
|  |  | Reverse primer | GCCTAATTTTTGTAATTCTTCATATTGG |  |
|  |  | Probe | CTATCCTGCTGACTTCTC |  |
| *asp23* | NWMN_2086 | Forward primer | CAAGCATACGACAATCAAACTGGTG | alkaline shock protein 23 |
|  |  | Reverse primer | GCAGCGATACCAGCAATTTTTTC |  |
|  |  | Probe | CGTCAAAAACAACAAGAAC |  |
| *atl* | NWMN_0922 | Forward primer | CGAAACAGCACCAACGGATTACTTA | autolysin |
|  |  | Reverse primer | CAGCATAGTTATTCATTGAACGTGCAA |  |
|  |  | Probe | ACTGCACCGACACCC |  |
| *aur* | NWMN_2536 | Forward primer | GAGCACTTTATCACCAGCAGCATTAG | zinc metalloproteinase aureolysin |
|  |  | Reverse primer | GTTTTTACATCAGTAACAGCGTAATCTTG |  |
|  |  | Probe | GAGGTGACTCAAAAGAG |  |
| *cap5H* | NWMN_0102 | Forward primer | GCTGAAAAACCAGTCCTCTAAAGAATC | capsular polysaccharide 5 |
|  |  | Reverse primer | CAAATCCAATATAACTGTATTCACCAATG |  |
|  |  | Probe | TAAGATTCATCGCTTGG |  |
| *capA* | NWMN_0095 | Forward primer | CAACTTATCAACATCCAAGTTAAAAGTGG | capsular polysaccharide synthesis enzyme CapA |
|  |  | Reverse primer | TTTGGTGCGACTTTAACTGCTG |  |
|  |  | Probe | CCGAAGATTATGAGTG |  |
| *capA2* | NWMN_2563 | Forward primer | GCCTGACAAATATACTGCTTCTACTC | capsular polysaccharide biosynthesis  protein capA2 |
|  |  | Reverse primer | GACTGCAAACTACTTTGAACATTTTGG |  |
|  |  | Probe | GTCCTCAAGTGATTTAG |  |

| **Assay name** | **Newman gene** | **Oligo function** | **Sequence** | **Function-notes** | | |
| --- | --- | --- | --- | --- | --- | --- |
| *chp* | NWMN_1877 | Forward primer | GGAATCAGTACACACCATCATTCAG | | chemotaxis-inhibiting protein CHIPS |  |
|  |  | Reverse primer | ATTTCTCAAACGTTCATCTAATTTTCC | |  |  |
|  |  | Probe | CCGTTTCCTACAAATG | |  |  |
| *clfA* | NWMN_0756 | Forward primer | CAACGAATCAAGCTAATACACCG | | clumping factor A, fibrinogen-binding protein A |  |
|  |  | Reverse primer | GTTGTTGAAACATTTTCCGCATTTG | |  |  |
|  |  | Probe | GTGAATCAAACAAGTAATG | |  |  |
| *clfB* | NWMN_2529 | Forward primer | GGATAGGCAATCATCAAGCACAAG | | clumping factor B |  |
|  |  | Reverse primer | GCTATCTACATTCGCACTGTTTGTG | |  |  |
|  |  | Probe | CAATATGATAGAAACACC | |  |  |
| *coa* | NWMN_0166 | Forward primer | GAAATAAAACCACAAGGTACTGAATCAACG | | staphylocoagulase |  |
|  |  | Reverse primer | GCTTCATATCCAAATGTTCCATCG | |  |  |
|  |  | Probe | CAATTTAACAAAACACC | |  |  |
| *eap* | NWMN_1872 | Forward primer | CGAAAATAAAGCTAAAAGAAACTATCAAG | | MHC class II analog protein |  |
|  |  | Reverse primer | GCTTTCTTAGCATATTTTAAATCTTGTTCAC | |  |  |
|  |  | Probe | CATTTTCAAATAAACCTTG | |  |  |
| *ebpS* | NWMN_1389 | Forward primer | AAAGGTGCAGCGATCGGT | | elastin binding protein |  |
|  |  | Reverse primer | GCGGCAGAAGCACTTTTACTTG | |  |  |
|  |  | Probe | CTCCAGCCAAACCTG | |  |  |
| *esaA* | NWMN_0220 | Forward primer | GTTGCTGAGTCTGGTTTGAAAAATGG | | essA, TVIISS |  |
|  |  | Reverse primer | GATATTTTCGATGGTGTTTTAGCGTC | |  |  |
|  |  | Probe | CATGATTGTTATCCCAG | |  |  |
| *esaB* | NWMN_0221 | Forward primer | CAGCACGTAAAAGTAACATTTGATTTTAC | | esaB, TVIISS |  |
|  |  | Reverse primer | TTTCAACAAGTAATTGACCTTTCGTC | |  |  |
|  |  | Probe | ATAATTACGGCACATATG | |  |  |
| *essA* | NWMN_0221 | Forward primer | CGACTCGCTTGAATGAAACTAAAAAAGTG | | essA, TVIISS |  |
|  |  | Reverse primer | CCCCTACAGACATCAAAATGTACG | |  |  |
|  |  | Probe | AAGACTTCGGAGAGTG | |  |  |

| **Assay name** | **Newman gene** | **Oligo function** | **Sequence** | **Function-notes** |
| --- | --- | --- | --- | --- |
| *essB* | NWMN_0222 | Forward primer | GATTCCTAAGTCTTCAATTAAACCAGAAC | essB, TVIISS |
|  |  | Reverse primer | GGTGTATGATTGTCATTAATGTCATAATG |  |
|  |  | Probe | CCATATTTTATAGATGCTG |  |
| *essC* | NWMN_0223 | Forward primer | TTCGCCAAGGATTATTCACCGTG | essC, TVIISS |
|  |  | Reverse primer | CTAATGGCGGTATAATGGAACG |  |
|  |  | Probe | CAATACAGAAGAACAATAC |  |
| *esxA* | NWMN_0219 | Forward primer | GAGTCCAGAGGAAATCAGAGCAAAA | esxA secreted protein |
|  |  | Reverse primer | CCTTGTGCACGTGTTAAATCAGATAAAA |  |
|  |  | Probe | CTTGCCCGTAAGATTG |  |
| *esxB* | NWMN_0225 | Forward primer | TCGCTGAGTATATCGAAGGTAGTGA | esxB secreted protein |
|  |  | Reverse primer | CGGTTGTACTAATTCTTCTTGAAACTTTGC |  |
|  |  | Probe | TTGGCGAACTGTCCTTC |  |
| *eta* | NWMN_1082 | Forward primer | GCTTTCTTGATTTGGATTCACCTTTTATG | exfoliative toxin A |
|  |  | Reverse primer | GCCAGACATGAAAAATGTTGTGAACAC |  |
|  |  | Probe | AATAATGTGAAAGAACAAT |  |
| *fbp* | NWMN_1119 | Forward primer | CACAAAATCAATCAACCTGATAATGAC | fibronectin/fibrinogen-binding protein |
|  |  | Reverse primer | AATCTTGAAAAGTTTGGATGGATTGAC |  |
|  |  | Probe | AAAATAGACAAAACCATC |  |
| *fhuD2* | NWMN_2185 | Forward primer | CCAACAGTAGTTGTTGACTATAATAAGCA | hydroxamate siderophore binding  lipoprotein |
|  |  | Reverse primer | GCAGTTGTTTCTTCCCAATCTTTCTT |  |
|  |  | Probe | CTTTACCAACAATTTTCC |  |
| *FLIPr* | NWMN_1067 | Forward primer | GCCAAGGTGATGTGAAGAAAGCAG | FLIPr |
|  |  | Reverse primer | CGAGTCGATTTCACCGTTTTTAACAAC |  |
|  |  | Probe | GTCCTTTAGAAGAGAACAG |  |
| *fnbA* | NWMN_2399 | Forward primer | ATTGAAACAATAGAAGAAACGGATTCATC | fnbA, C-term truncation in Newman |
|  |  | Reverse primer | CTTCAAAGTCAATTGGATTTGATTCCTC |  |
|  |  | Probe | CCATACTGCTGTGGATAG |  |

| **Assay name** | **Newman gene** | **Oligo function** | **Sequence** | **Function-notes** |
| --- | --- | --- | --- | --- |
| *fnbB* | NWMN_2397 | Forward primer | GCGACATCAACTGAGCAACCATC | fnbB, C-term truncation in Newman |
|  |  | Reverse primer | GAAGTTTCTACTTTTGGTGCTTGCACAG |  |
|  |  | Probe | TAACAACAGAAGAAGCAC |  |
| *geh* | NWMN_0262 | Forward primer | CACATCAAATGCAGTCAGGAAAGC | triacylglycerol lipase |
|  |  | Reverse primer | CTTGTCGTTCAGAATCTTGCTTTACTTG |  |
|  |  | Probe | GTGGAACAGTGACAGAAG |  |
| *gyrB* | NWMN_0004 | Forward primer | GGTGACTGCATTGTCAGATGTAAAC | DNA gyrase subunit B |
|  |  | Reverse primer | CTGCTTCTAAACCTTCTAATACTTGTATTTG |  |
|  |  | Probe | CCCAGCACCATAATTA |  |
| *hla* | NWMN_1073 | Forward primer | TATAGTCAGCTCAGTAACAACAACA | alpha-hemolysin precursor |
|  |  | Reverse primer | TGCATGCCATTTTCTTTATCATAAGTGAC |  |
|  |  | Probe | ATGCCGCAGATTCT |  |
| *hlb* | NWMN_1926 | Forward primer | GTAATATTCAATGAAGCATTTGATAATGG | beta-hemolysin |
|  |  | Reverse primer | GGATATTTACTTACAATCGCTACGCC |  |
|  |  | Probe | CTGAAGGTAGCTACTCATC |  |
| *hld* | NWMN_2624 | Forward primer | TTAAGGAAGGAGTGATTTCAATGG | delta-hemolysin, RNAIII |
|  |  | Reverse primer | TGAATTTGTTCACTGTGTCGATAATC |  |
|  |  | Probe | GATATCATTTCAACAATC |  |
| *hlgA* | NWMN_2318 | Forward primer | GCAGAAAATAAGATAGAAGATATCGG | gamma-hemolysin component A |
|  |  | Reverse primer | TCAAATTGAATGTTTTGAGTTATAGC |  |
|  |  | Probe | GTGCAGAAATCATCAAAAG |  |
| *hlgB2* | NWMN_2320 | Forward primer | GCTACTGGGAATATTAACTCAGGCTTTG | gamma hemolysin, component B |
|  |  | Reverse primer | GTGCATAATCAACGACGTTTACTGAATC |  |
|  |  | Probe | GGGAGCTAAATACAATG |  |
| *hlgC* | NWMN_2319 | Forward primer | GATACTGAAGACATCGGTAAAGGAA | gamma-hemolysin component C |
|  |  | Reverse primer | AGAGCTAATGAATCCTTGCATCTTTAA |  |
|  |  | Probe | TTATCAAAAGGACAGAAG |  |

| **Assay name** | **Newman gene** | **Oligo function** | **Sequence** | **Function-notes** |
| --- | --- | --- | --- | --- |
| *hlgC2* | NWMN_2319 | Forward primer | GATAAAACAAGTAATAAATGGGGCGTGAC | gamma-hemolysin component C |
|  |  | Reverse primer | GAATGGCCATCGCATAGCTTTAAC |  |
|  |  | Probe | GATATTAAAGATGCAAGG |  |
| *hysA* | NWMN_2106 | Forward primer | GGAATGGATTTTGAAAATCAGGAC | hyaluronate lyase |
|  |  | Reverse primer | GATGAATCAGTACTTTTAATGCCAGTTCC |  |
|  |  | Probe | CATATTTCATATTAAACG |  |
| *icaB* | NWMN_2567 | Forward primer | GGATGGTCATCATATTGCAAATGCA | intercellular adhesion protein IcaB |
|  |  | Reverse primer | AATTCGCTTTTCTTACACGGTGATAATTT |  |
|  |  | Probe | CCAGAGCACTATTTTC |  |
| *isaA* | NWMN_2469 | Forward primer | CGTTGATCAAGCACACTTAGTTGACTTAG | immunodominant antigen A |
|  |  | Reverse primer | GCTCCATGACCATGTAGTACCATTTGAAG |  |
|  |  | Probe | CTCCAATCAAAGATGGTG |  |
| *isaB* | NWMN_2537 | Forward primer | GGCAAGGACTTGAAAAAAGAAAATGGT | immunodominant antigen B |
|  |  | Reverse primer | CGACAACTCTATTATGATCAACGACAAAC |  |
|  |  | Probe | ACCGCTATCAGCTTCC |  |
| *isdA* | NWMN_1041 | Forward primer | GCAGTTGAACCTGGATATAAGAGCTTA | iron-regulated heme-iron binding  protein |
|  |  | Reverse primer | TGCTTTTTCAAATTCCAAATGCGTAGT |  |
|  |  | Probe | TCGTGCCACAAATTAA |  |
| *isdB* | NWMN_1040 | Forward primer | GGAGAAAATTTGAAGTTTATGAAGGTGACA | iron-regulated heme-iron binding  protein |
|  |  | Reverse primer | TGTTTTCGCTTTTTTATATGGCGCTAA |  |
|  |  | Probe | CAGTGCAGATAAATTC |  |
| *isdC* | NWMN_1042 | Forward primer | TAATTATCATCATCGCGACATTCAG | iron-regulated cell surface protein |
|  |  | Reverse primer | CCATTTTTCTTAATGTACTTTGCCGG |  |
|  |  | Probe | CAATACCAATGACACGTC |  |
| *isdG* | NWMN_1047 | Forward primer | CGAGACATGGGATTGAAACATTAGAAG | cytoplasmic heme-iron binding protein |
|  |  | Reverse primer | GGGCTACTTTCATCTTCATTTTTACTTC |  |
|  |  | Probe | ACAGTTTGGAAATCAAA |  |

| **Assay name** | **Newman gene** | **Oligo function** | **Sequence** | **Function-notes** |
| --- | --- | --- | --- | --- |
| *isdH* | NWMN_1624 | Forward primer | GTTGCATCGGTCATTGTCAGTAC | haptoglobin-binding surface anchored  protein |
|  |  | Reverse primer | GTTGCATTATTATTTTGATTTTCCG |  |
|  |  | Probe | CAAGCAGCAGAAAATAC |  |
| *ltaA* | NWMN_0886 | Forward primer | CTGTAGCAATAACGTCTCTAGCATTTTC | glycolipid permease LtaA |
|  |  | Reverse primer | GGTGATGCTGGAAACCAAATAAC |  |
|  |  | Probe | CAAAAATCGTTTTAACG |  |
| *ltaS* | NWMN_0687 | Forward primer | CCGTAATAACGATTACCTTGAAGACG | glycerol phosphate lipoteichoic acid  synthase |
|  |  | Reverse primer | TTTTTTGCCTTTAAAGAATAGGAACACAC |  |
|  |  | Probe | TGAATCCTTATAGTTTAG |  |
| *lukD* | NWMN_1718 | Forward primer | GAAAGTTACAGAACTACGATTGATAGAAAAACA | leukocidin LukD subunit |
|  |  | Reverse primer | ATTATTCATAATTTTGTGCGCCTCAACA |  |
|  |  | Probe | CCCCAGCCAATTGA |  |
| *lukE* | NWMN_1719 | Forward primer | GATGTTGGTCAAACATTAGGATATAACATTG | leukocidin LukE subunit |
|  |  | Reverse primer | ATTGTTTTAGAATAATTAAATGAGCCATTGCCA |  |
|  |  | Probe | CTGACTGGAAATTACC |  |
| *lukS* | NWMN_1928 | Forward primer | GCAGCAACGACTCAAGCAAATTC | leukocidin LukS subunit |
|  |  | Reverse primer | GTTTCAGTTCGTTTTGTGATTTTACCG |  |
|  |  | Probe | GAACATGTTGATAAGTCTC |  |
| *mgrA* | NWMN_0655 | Forward primer | GCTCAAAGACAAGTTAATCGCTACTACTC | MgrA - MarR family regulatory protein |
|  |  | Reverse primer | GTGCTAATTCAGTTACGACTTTCTTGAC |  |
|  |  | Probe | CCCACAATTTCTTGTC |  |
| *mntA* | NWMN_0603 | Forward primer | GTGTGGAACAAGTGATTTTATCAGG | ABC transporter ATP-binding protein |
|  |  | Reverse primer | CACTTAATTCTGAAATTTGTCGATGAC |  |
|  |  | Probe | GGATGGTTTAGACGACC |  |
| *mntH* | NWMN_0971 | Forward primer | GGAAACTGGATAACATCAATGCAAG | Mn2+/Fe2+ transporter NRAMP  family protein |
|  |  | Reverse primer | GTCGTGTCATTTGAGCTAAGTCCATAC |  |
|  |  | Probe | GGCTATACTTTGCTATTC |  |

| **Assay name** | **Newman gene** | **Oligo function** | **Sequence** | **Function-notes** |
| --- | --- | --- | --- | --- |
| *nuc* | NWMN_1236 | Forward primer | CCTGTACAACCATTTGGCAAAGAAGC | thermonuclease |
|  |  | Reverse primer | GCAAGTCCCTTTTCCACTAATTCC |  |
|  |  | Probe | CGCTATGGTAGAACATTG |  |
| *NWMN_0677* | NWMN_0677 | Forward primer | GTGAAACTGTTGAAGGTAAAGCTG | hypothetical protein |
|  |  | Reverse primer | ACCATTGCGATTTCTTTACC |  |
|  |  | Probe | CAAATCATCAAAAGGTCC |  |
| *NWMN_0851* | NWMN_0851 | Forward primer | CAATTGCAGTAGATGGCATTATGGC | putative surface protein |
|  |  | Reverse primer | CTTTCCAAGTAATCGTGTAAACGGCAG |  |
|  |  | Probe | CCAAAAGATAGCCAATTA |  |
| *NWMN_1231* | NWMN_1231 | Forward primer | GGTGCTGGAAAGTCAACGTTAATTG | ABC transporter (ATP-binding protein)  homolog |
|  |  | Reverse primer | GAAACATCGTTTTTTGGAACATTATACTG |  |
|  |  | Probe | AATTCTGGTGAGATATTTG |  |
| *saeP* | NWMN_0677 | Forward primer | CGTAGTCAACCATTGCGATTTC | SaeRS auxiliary protein |
|  |  | Reverse primer | GTGAAACTGTTGAAGGTAAAGCTGAG |  |
|  |  | Probe | GACGTATAAATCTGGACC |  |
| *sak* | NWMN_1880 | Forward primer | GAGGTAAGTGCATCAAGTTCATTCGAC | staphylokinase |
|  |  | Reverse primer | GTCCCAGGTTTAATAGGAAACTCGAC |  |
|  |  | Probe | GATGGTAAATGTGACTGG |  |
| *sarA* | NWMN_0588 | Forward primer | GAGTTGTTATCAATGGTCACTTATGCTG | SarA transcriptional regulator |
|  |  | Reverse primer | CTTTGTTTTCGCTGATGTATGTCAATAC |  |
|  |  | Probe | GAATTTTCAATTAGCTTTG |  |
| *sarR* | NWMN_2195 | Forward primer | GTCAACGCAACATTTCAAGTTAAGAAG | SarR transcriptional regulator |
|  |  | Reverse primer | CTCTGAGCACTTAGCAATCTCTTTAGATG |  |
|  |  | Probe | TCAATTTGAACTATGAAG |  |
| *sarS* | NWMN_0056 | Forward primer | GATGAGCGTAATACTTACATTTCAATATCTG | SarS transcriptional regulator |
|  |  | Reverse primer | CTATCTTTTGGTATCATCTGTGATTCAC |  |
|  |  | Probe | CAGAACGTGTTACATTG |  |

| **Assay name** | **Newman gene** | **Oligo function** | **Sequence** | **Function-notes** |  |
| --- | --- | --- | --- | --- | --- |
| *sarZ* | NWMN_2286 | Forward primer | GGTTACATTGTTTTAATGGCGATTG | SarZ - MarR family regulatory protein | |
|  |  | Reverse primer | CATCTTTCTCTTCACGTGTTCGAAC |  |  |
|  |  | Probe | CTTAGATTCTGGAACACTG |  |  |
| *sasA* | NWMN_2553 | Forward primer | GCGACAAATTTACAACAAGTACAATTTGG | serine-threoinine rich antigen | |
|  |  | Reverse primer | CGATTGTCACGACTTGATCAACATTTC |  |  |
|  |  | Probe | CTGCTGTTACACAAGTG |  |  |
| *sasB* | NWMN_2061 | Forward primer | CTACTATGCAAACGAATAGTAAGCAAGG | methicillin resistance determinant  FmtB protein | |
|  |  | Reverse primer | GTAATTCTTGAAGCATCAGCAACTGC |  |  |
|  |  | Probe | GAATTAGCAACTGTAAATG |  |  |
| *sasC* | NWMN_1649 | Forward primer | GGTTCAGGAGGACATCTAACTTTAAAGG | similar to fmtB protein, cell wall anchored | |
|  |  | Reverse primer | TGCCGCACGTCTACTTCTCTTTTTC |  |  |
|  |  | Probe | GAGCTAGTTGCAATTGC |  |  |
| *sasD* | NWMN_0078 | Forward primer | CCTTATGGCGGAGTAGTACCACAAG | similar to functionally unknown protein | |
|  |  | Reverse primer | GCGTCGCATCATACAATTTCATATTATAG |  |  |
|  |  | Probe | GCACAATATACTGAATTAG |  |  |
| *sasF* | NWMN_2545 | Forward primer | CATTGATTGATCAATCACAAGATAAGTCG | similar to functionally unknown protein | |
|  |  | Reverse primer | CGATTTGATAATCCTTTATTCGTCC |  |  |
|  |  | Probe | TTACAAACGAAATTAGG |  |  |
| *sasG* | NWMN_2392 | Forward primer | CGAGAAAATACCGCAAGGTCATAAA | cell wall surface anchor family protein | |
|  |  | Reverse primer | TGTCTGGATTCTTGATTCCTGGTTT |  |  |
|  |  | Probe | CAGATCAAACGGAAAAAGTA |  |  |
| *sbi* | NWMN_2317 | Forward primer | GAAGAACAACGTAACCAATACATCAAAAC | immunoglobulin G-binding protein Sbi | |
|  |  | Reverse primer | GTAAAAAGCGTTTTGTTGTGCAACAC |  |  |
|  |  | Probe | GAAGTATTCTCTGAATCAC |  |  |
| *scn* | NWMN_1876 | Forward primer | CTTGCCAACATCGAATGAATATCAAAAC | staphylococcal complement inhibitor  SCIN | |
|  |  | Reverse primer | GTCTTTTGACTTAAGAGCATACATTGC |  |  |
|  |  | Probe | GATGAACTAAATGTTAATG |  |  |

| **Assay name** | **Newman gene** | | **Oligo function** | | | **Sequence** | | **Function-notes** |
| --- | --- | --- | --- | --- | --- | --- | --- | --- |
| *scpA* | NWMN_1403 | | Forward primer | | | CAGAGCAGTATATGCAGTACGTTCATGC | | Staphopain A, cystein protease |
|  |  |  | Reverse primer | | | GATATTCTATTAAACGCCCAACTAAATC | |  |
|  |  |  | Probe | | | TACCACAATCAACATCAG | |  |
| *sdrC* | NWMN_0523 | | Forward primer | | | ATGAATAATAAAAAGACAGCAACAAATAGA | | Ser-Asp rich fibrinogen/bone  sialoprotein-binding protein SdrC |
|  |  |  | Reverse primer | | | AGCAGTACCTACAGAATACTTTCTTATCGA | |  |
|  |  |  | Probe | | | AAAGGCATGATACCAAATCG | |  |
| *sirA* | NWMN_0059 | | Forward primer | | | CATGGACACAAAAACCGAAATTC | | siderophore compound ABC transporter  binding protein |
|  |  |  | Reverse primer | | | CATTTCTAACTTTTGACGCGACAATTAAG | |  |
|  |  |  | Probe | | | CTAAGATTGTAGGTCAAG | |  |
| *spa* | NWMN_0055 | | Forward primer | | | CAAACCTGGTCAAGAACTTGTTGTTG | | Immunoglobulin G binding protein A |
|  |  |  | Reverse primer | | | GCTAATGATAATCCACCAAATACAGTTG | |  |
|  |  |  | Probe | | | CATGCAGATGCTAAC | |  |
| *sspA* | NWMN_0918 | | Forward primer | | | CTTATATTCAAGTTGAAGCACCTACTGG | | V8 protease, glutamyl endopeptidase  precursor |
|  |  |  | Reverse primer | | | CTTTTAAAGCATGAGGATCACCGTG | |  |
|  |  |  | Probe | | | GGTGTAGTTGTAGGTAAAG | |  |
| *sspB* | NWMN_0917 | | Forward primer | | | CAACAACAATTTGCTGGTTATGCTAAAG | | cysteine protease, staphylopain |
|  |  |  | Reverse primer | | | TAAACAATTTTACCGTCTTTTATAACTGG | |  |
|  |  |  | Probe | | | GTAATGCAAAAACTGG | |  |
| *csa1A* | NWMN_0042 | | Forward primer | | | GAAGATATGGTAGCTAAAGGCATGGTTC | | staphylococcal tandem lipoprotein |
|  |  |  | Reverse primer | | | GTGCGGTTTTCCTTCATCC | |  |
|  |  |  | Probe | | | CTACTATGTCGATGTGACT | |  |
| *csa2A* | NWMN_0148 | | Forward primer | | | CGCTTTATGAAATTGATGGTCACG | | staphylococcal tandem lipoprotein |
|  |  |  | Reverse primer | | | CTTATCTACCTTTAACAACATACGGTCTTC | |  |
|  |  |  | Probe | | | CAGTTGTTGGATCAGATG | |  |
| *csa3A* | NWMN_0403 | | Forward primer | | | GAACCGCTAAAGGCCATTATTTTGTTAC | | staphylococcal tandem lipoprotein |
|  |  |  | Reverse primer | | | CTGCTTGAGCTTATCATCTTTTACTTC | |  |
|  |  |  | Probe | | | CCGGAATGGTAAACTAC | |  |
| *csa4A* | | NWMN_2379 | | Forward primer | GAACCACAAAAGGGTATTATTTTATAAGTG | | staphylococcal tandem lipoprotein | |
|  |  |  |  | Reverse primer | CTTGTCATTCGGTAGTGGCTTCG | |  |  |
|  |  |  |  | Probe | GTAACGGCAGACCAAAG | |  |  |
| *vWbp* | | NWMN_0757 | | Forward primer | GAAAAATAAATTGCTAGTTTTATCATTGG | | secreted von Willebrand factor-binding  protein | |
|  |  |  |  | Reverse primer | TCCCCAGAAACCACTGCAC | |  |  |
|  |  |  |  | Probe | GTGTATCACAAATTTGG | |  |  |
